# Supplementary figures and images for: Role of the Hedgehog Pathway and CAXII in Controlling Melanoma Cell Migration and Invasion in Hypoxia
Source: Cancers (Basel). 2022 Sep 29;14(19):4776. doi: 10.3390/cancers14194776 (PMC9563772; doi:10.3390/cancers14194776)

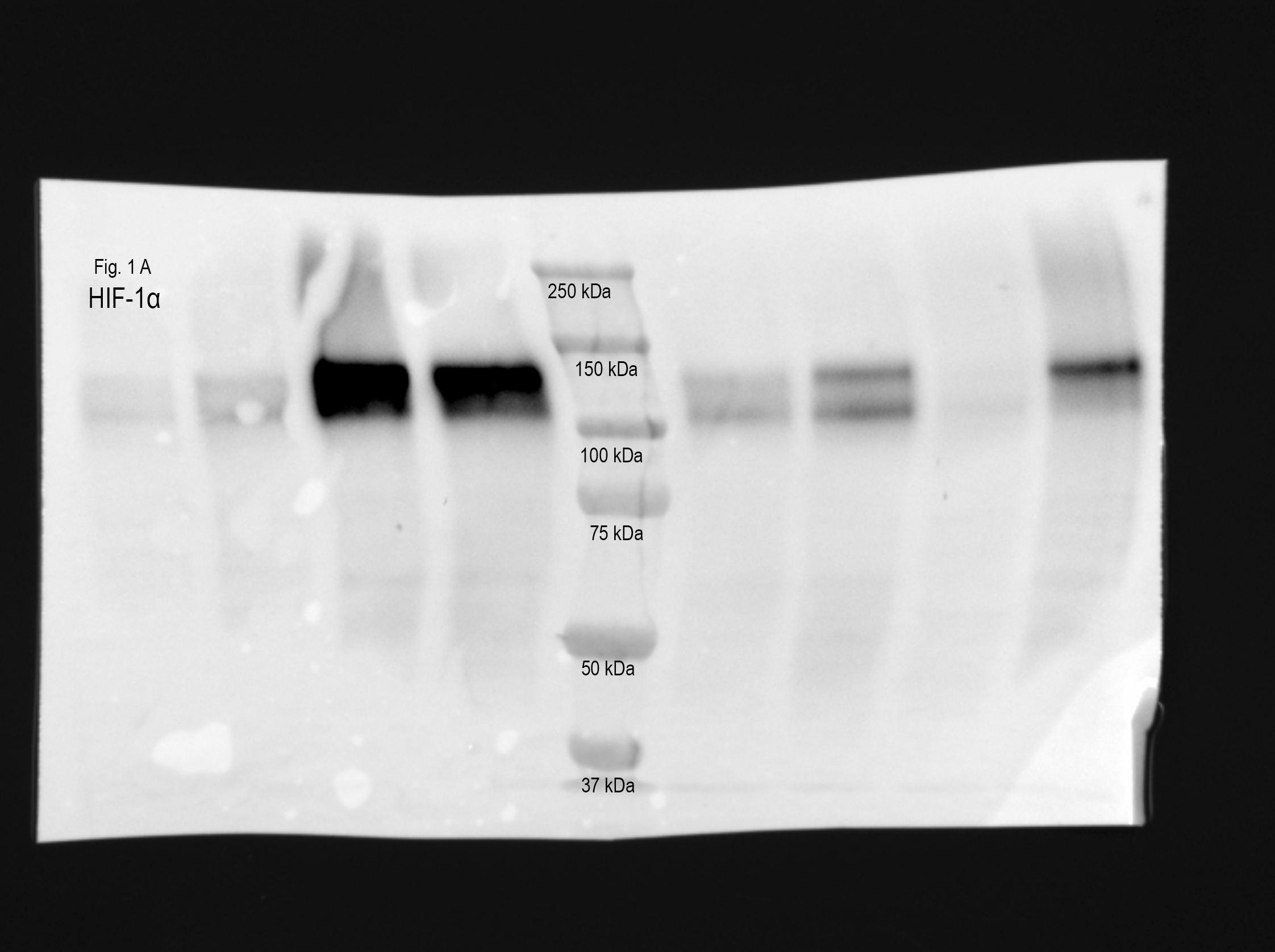

Supplement: Supplementary file 1 [file cancers-14-04776-s001.zip › File S1-blot jpg/0. HIF.jpg]

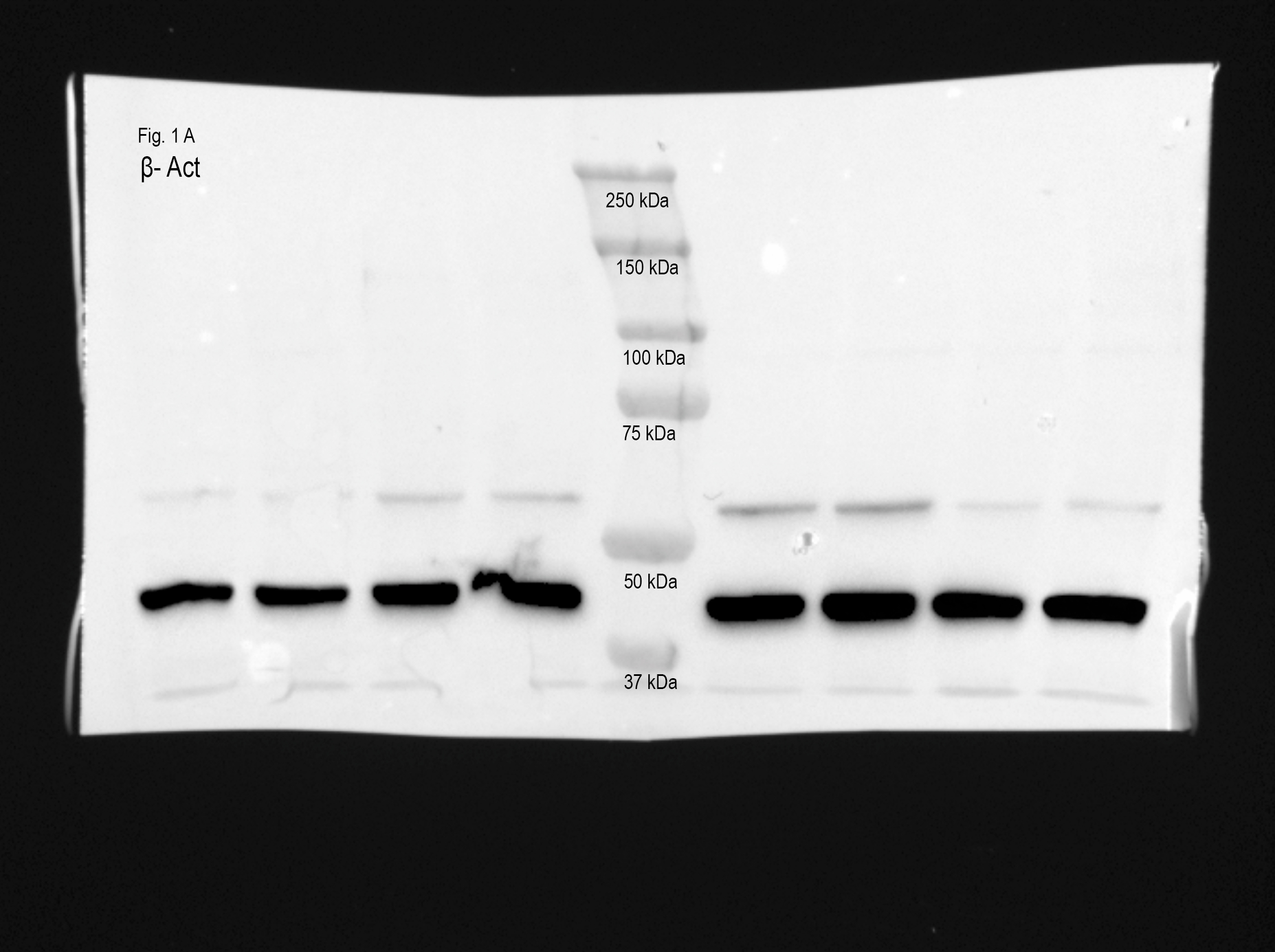

Supplement: Supplementary file 1 [file cancers-14-04776-s001.zip › File S1-blot jpg/1. HIF BACT.jpg]

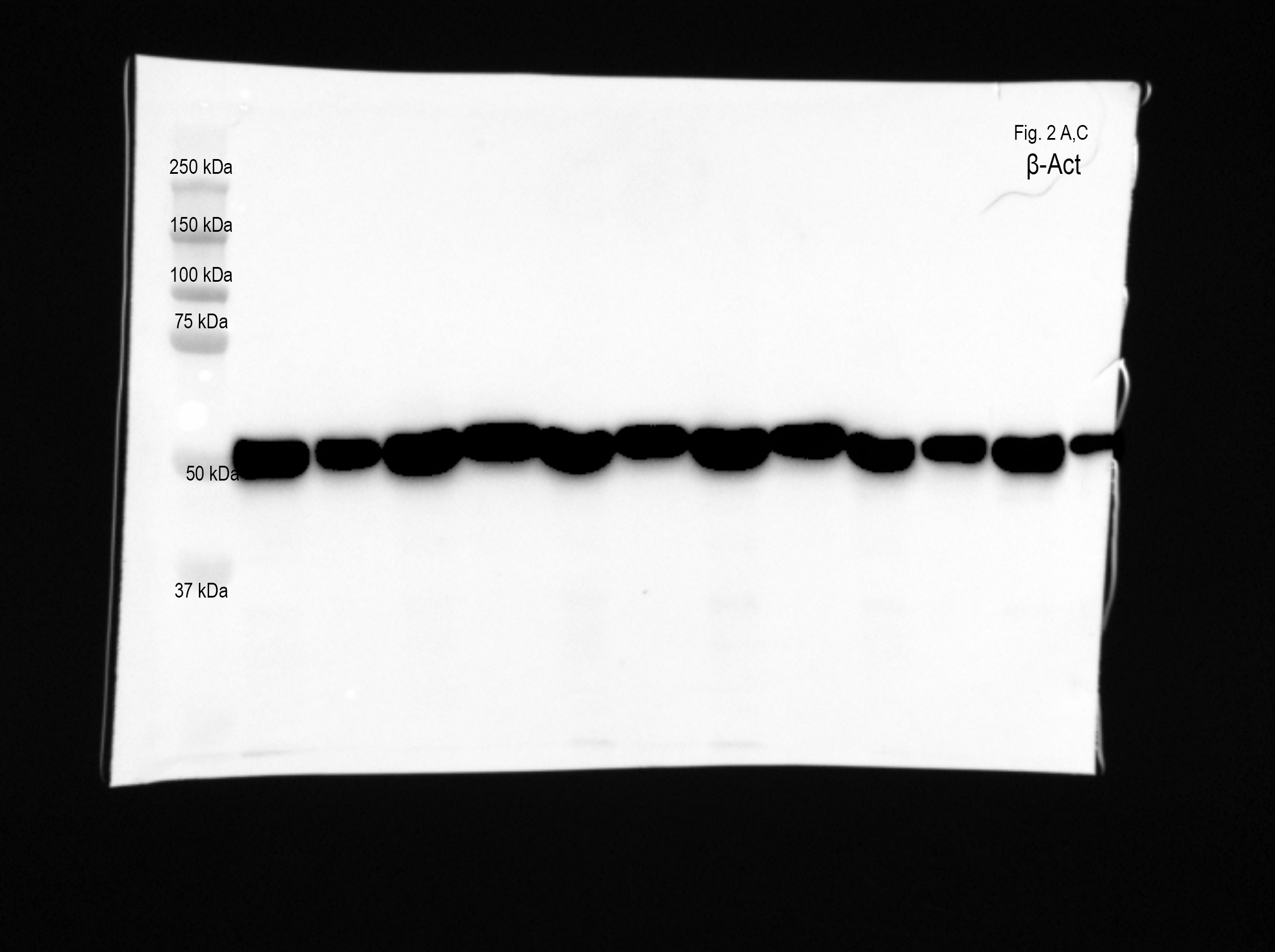

Supplement: Supplementary file 1 [file cancers-14-04776-s001.zip › File S1-blot jpg/10. FIG 2A SKMEL siSMO siGLI CAXII BACT.jpg]

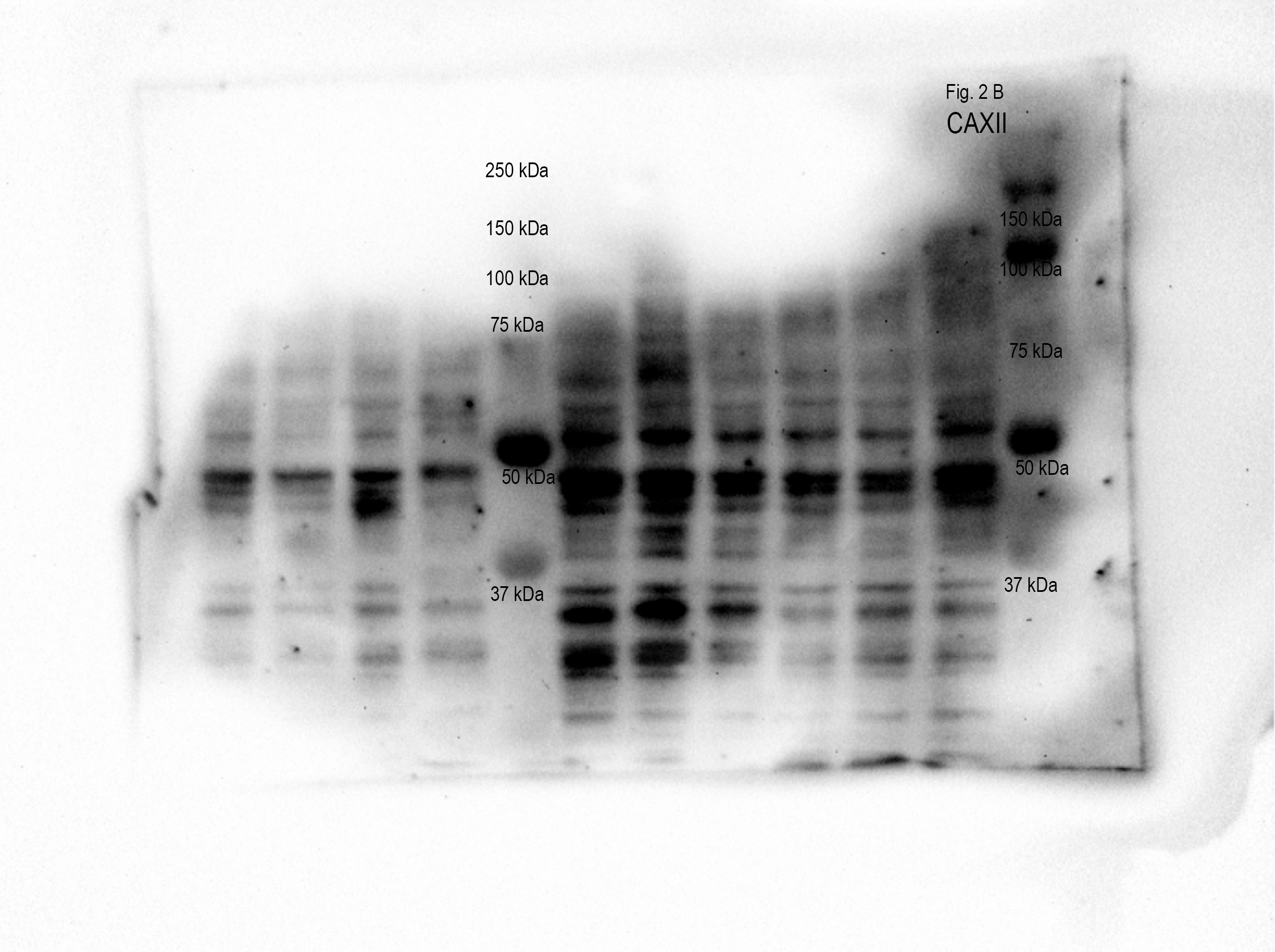

Supplement: Supplementary file 1 [file cancers-14-04776-s001.zip › File S1-blot jpg/11. A375 siSMO CAXII.jpg]

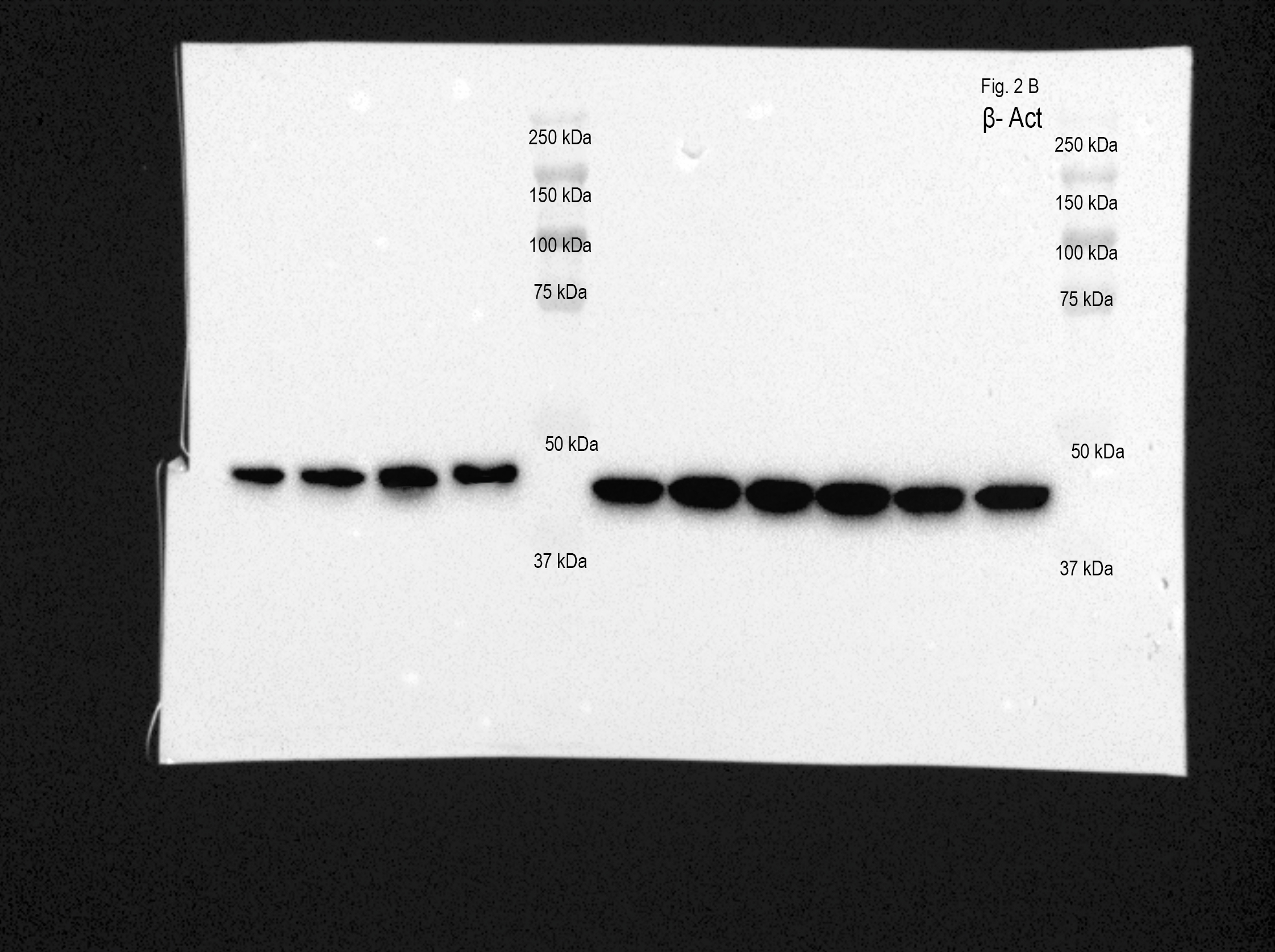

Supplement: Supplementary file 1 [file cancers-14-04776-s001.zip › File S1-blot jpg/12. A375 siSMO CAXII BACT.jpg]

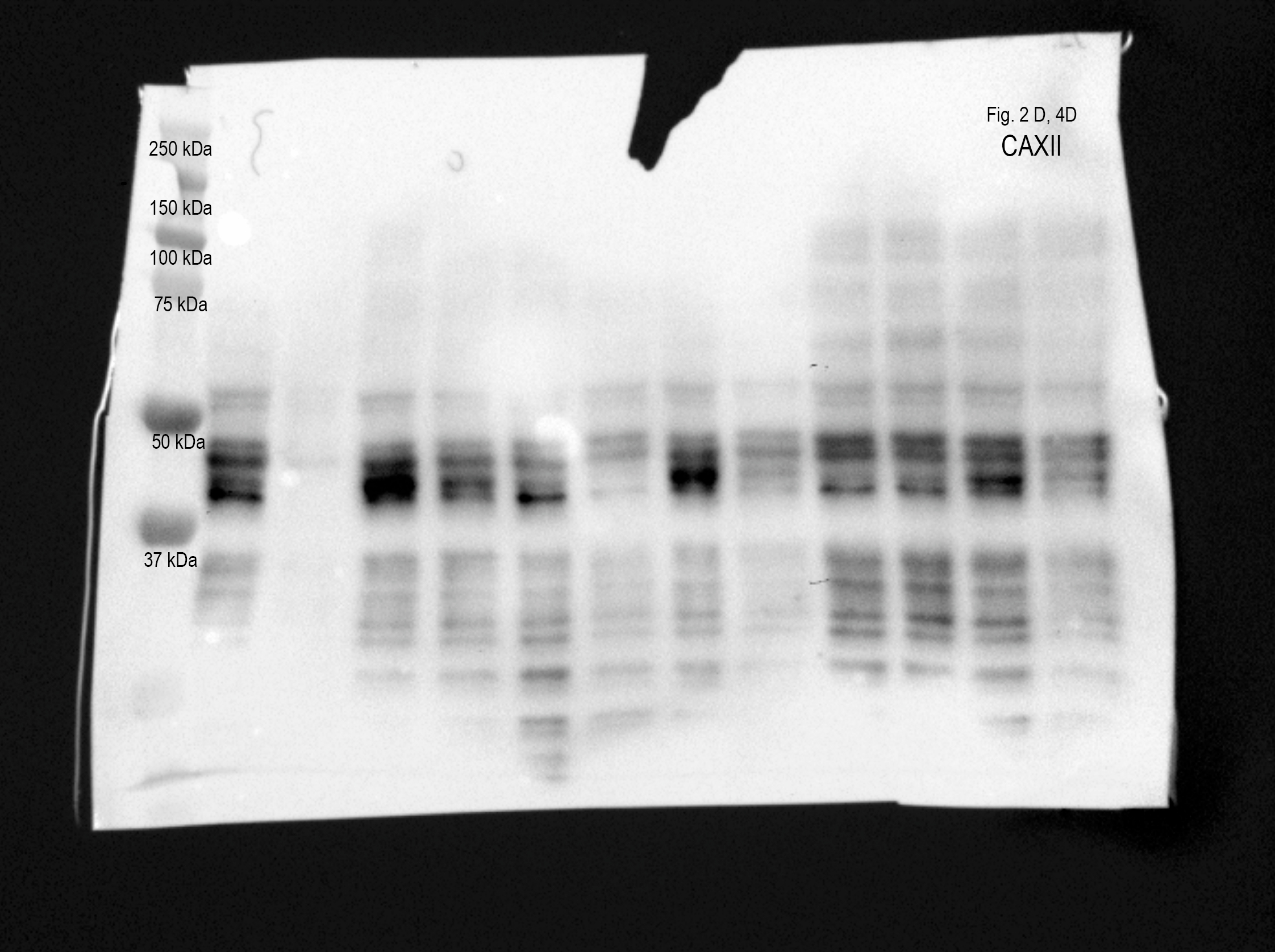

Supplement: Supplementary file 1 [file cancers-14-04776-s001.zip › File S1-blot jpg/13. A375 siGLI e siCAXII CAXII.jpg]

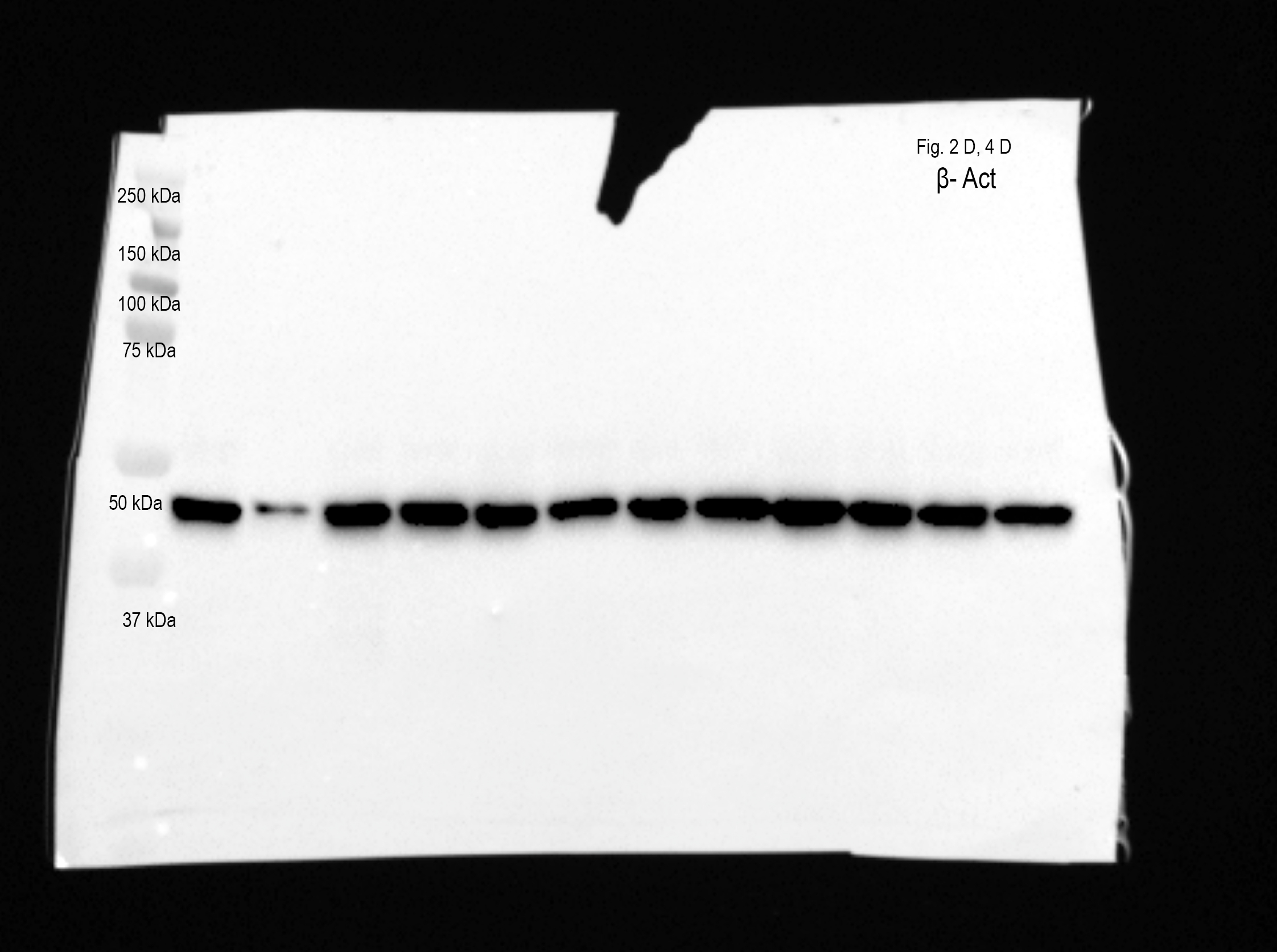

Supplement: Supplementary file 1 [file cancers-14-04776-s001.zip › File S1-blot jpg/14. A375 siGLI e siCAXII CAXII BACT.jpg]

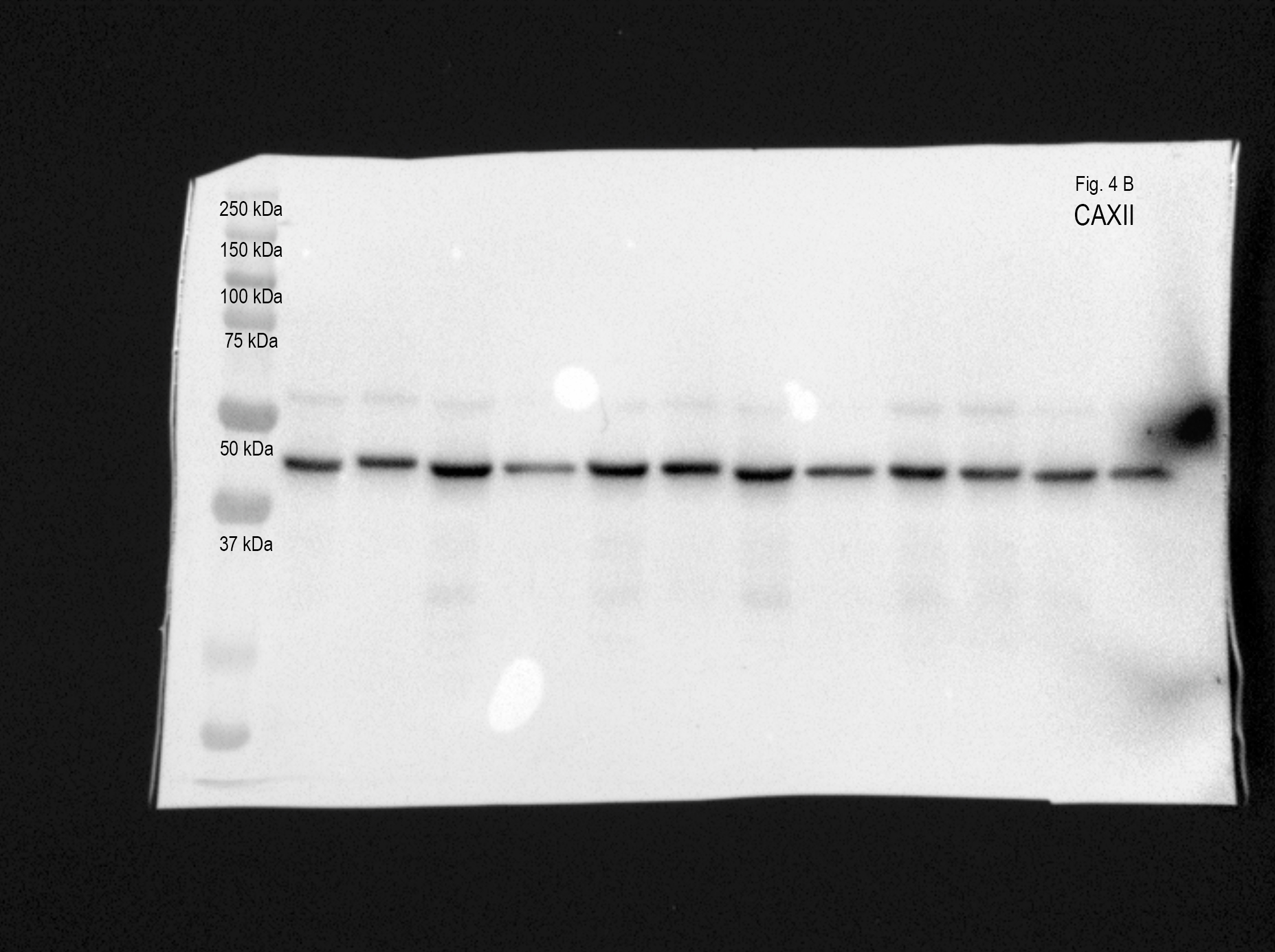

Supplement: Supplementary file 1 [file cancers-14-04776-s001.zip › File S1-blot jpg/15. SKMEL siCAXII CAXII.jpg]

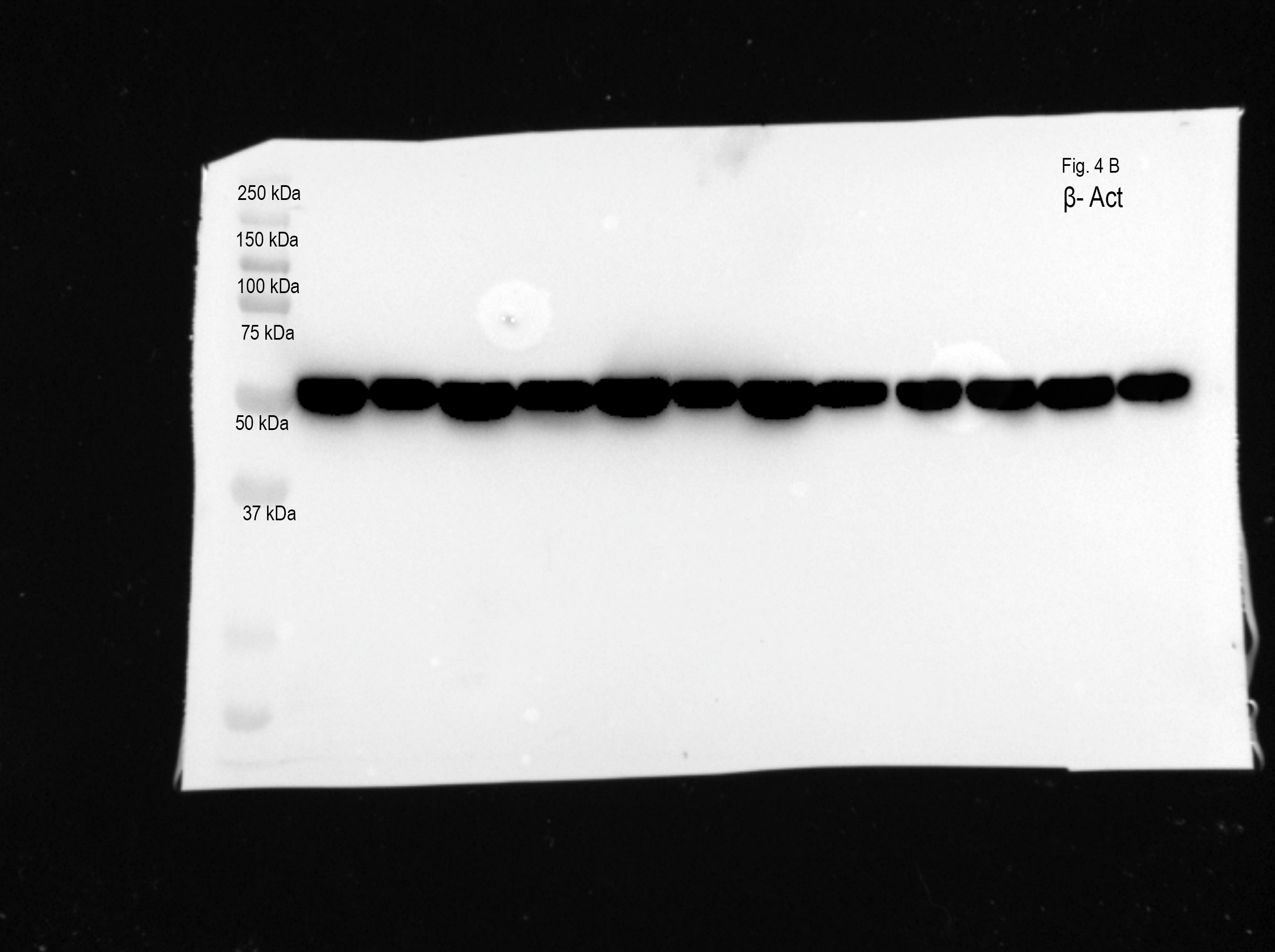

Supplement: Supplementary file 1 [file cancers-14-04776-s001.zip › File S1-blot jpg/16. SKMEL siCAXII CAXII BACT.png]

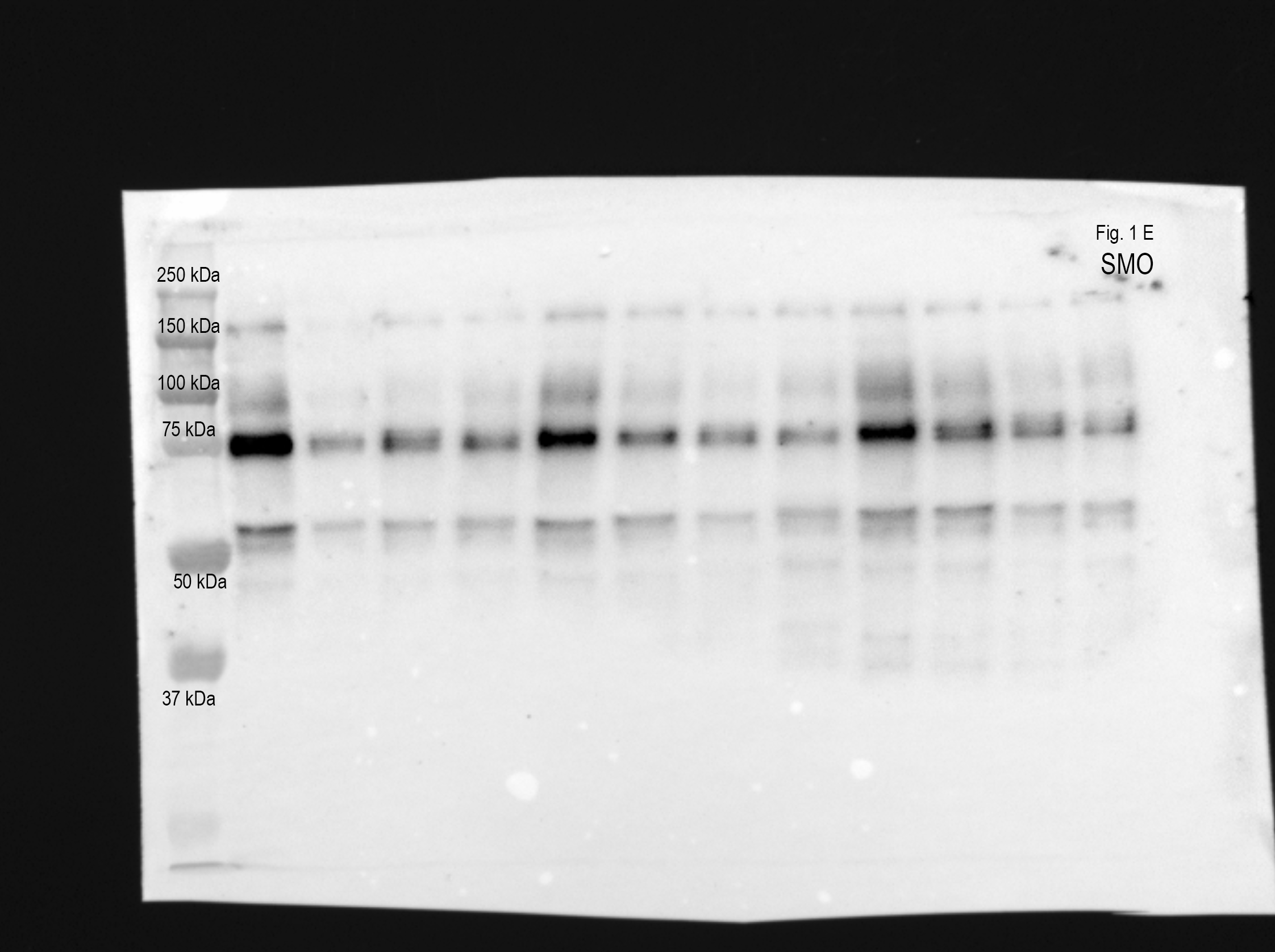

Supplement: Supplementary file 1 [file cancers-14-04776-s001.zip › File S1-blot jpg/17 A375 siSMO SMO.tif]

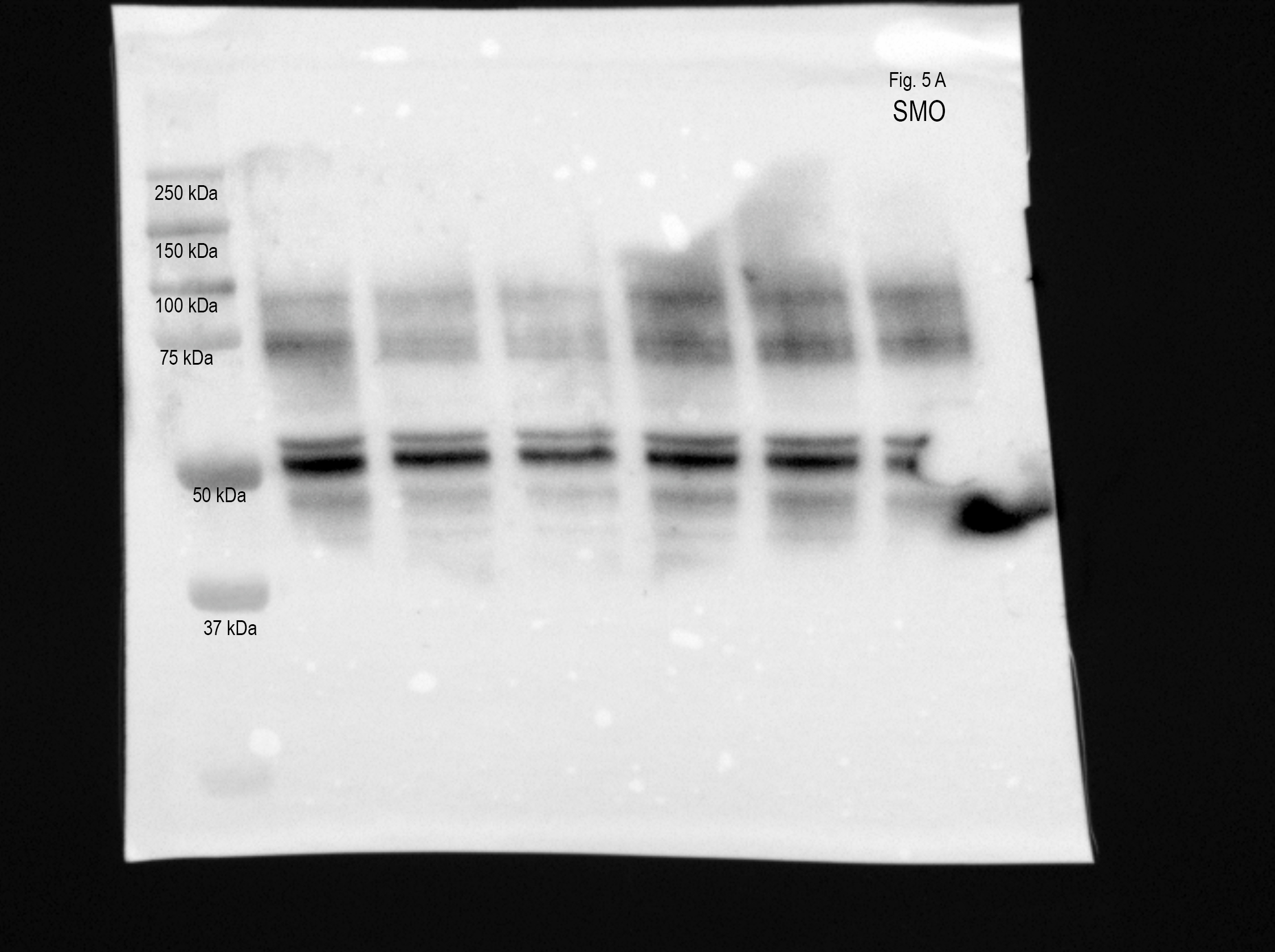

Supplement: Supplementary file 1 [file cancers-14-04776-s001.zip › File S1-blot jpg/17. SKMEL INIBITORI SMO.jpg]

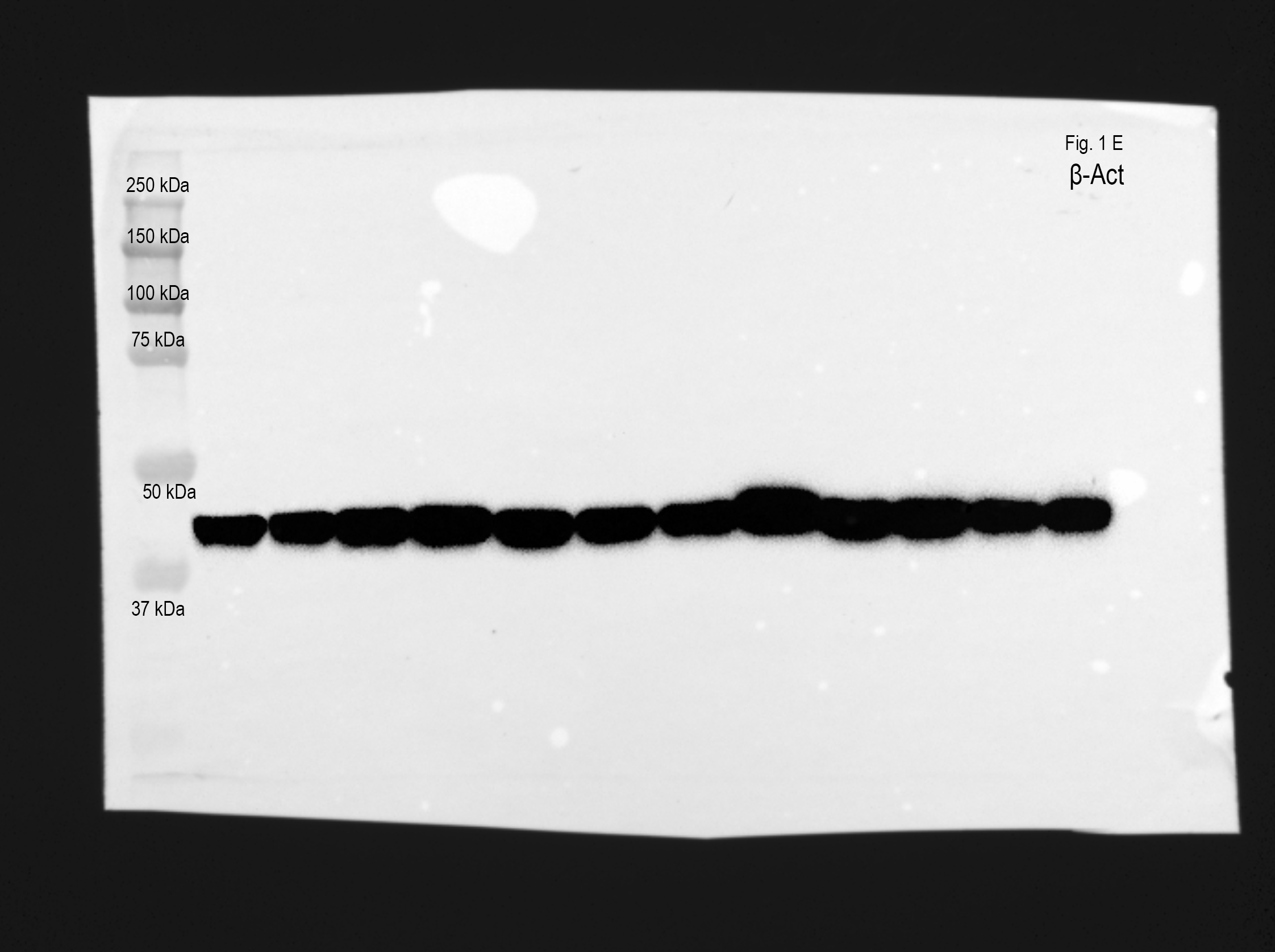

Supplement: Supplementary file 1 [file cancers-14-04776-s001.zip › File S1-blot jpg/18 A375 siSMO bact.tif]

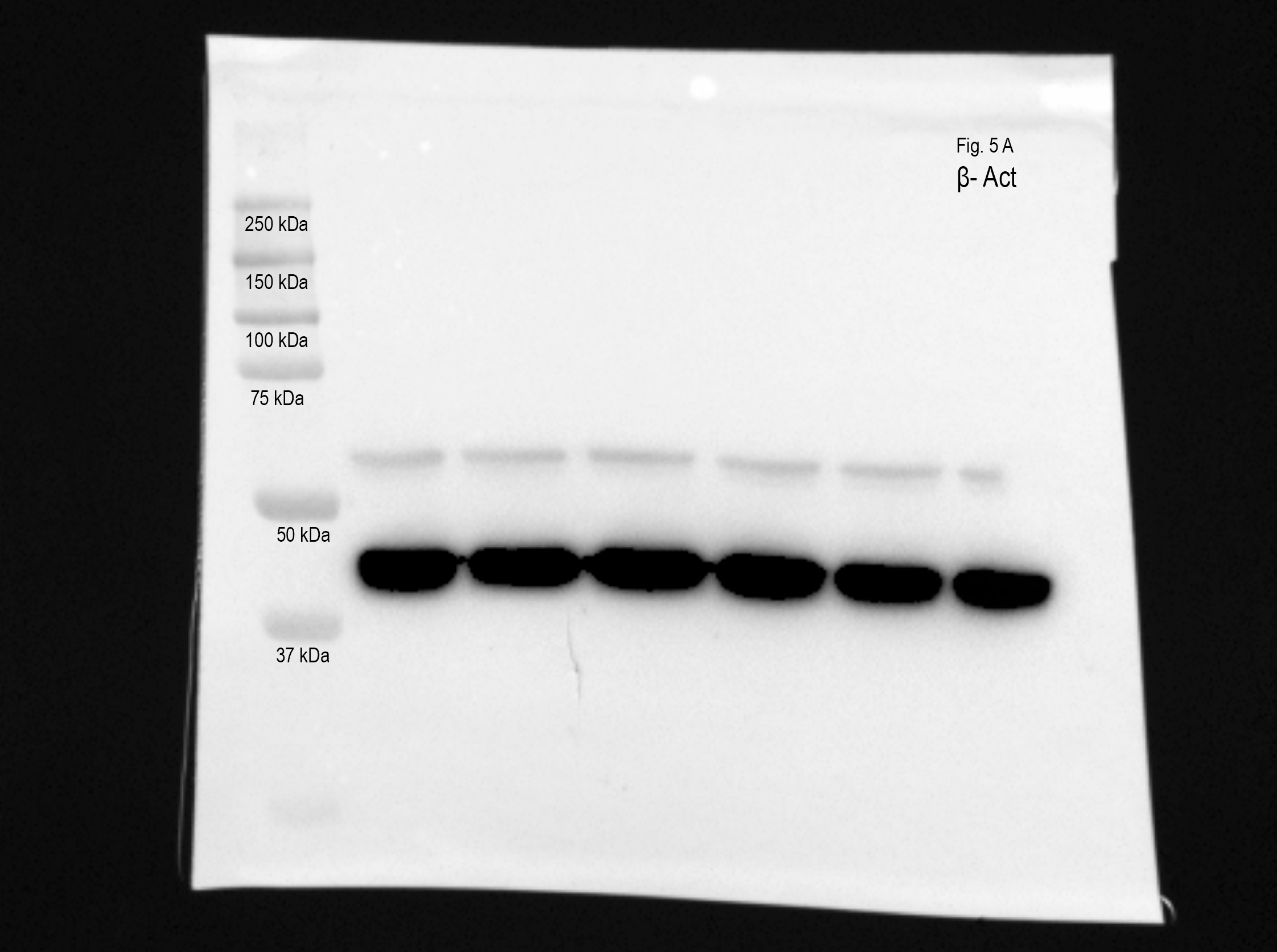

Supplement: Supplementary file 1 [file cancers-14-04776-s001.zip › File S1-blot jpg/18. SKMEL INIBITORI SMO BACT.jpg]

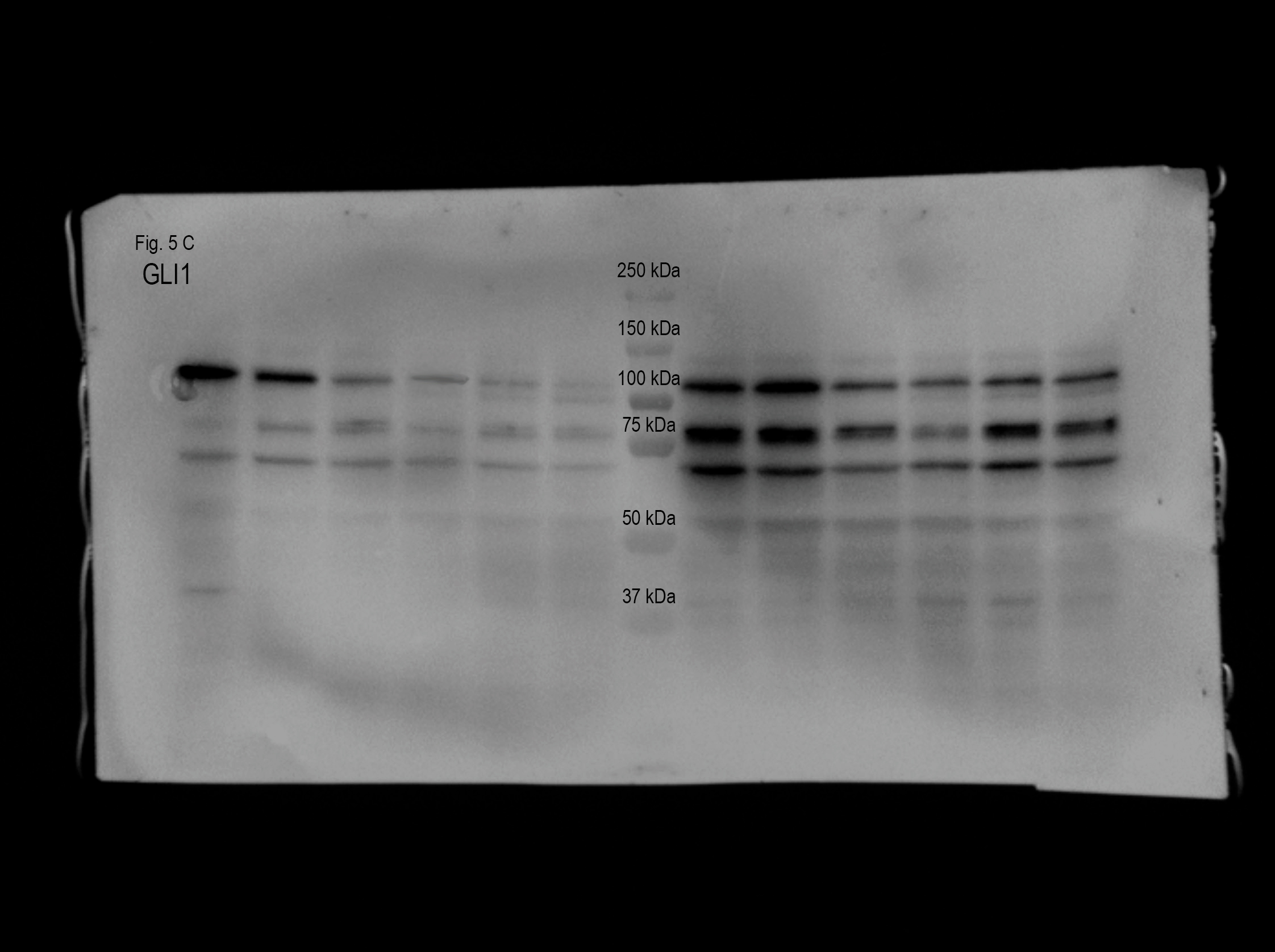

Supplement: Supplementary file 1 [file cancers-14-04776-s001.zip › File S1-blot jpg/19. SKMEL INIBITORI GLI.jpg]

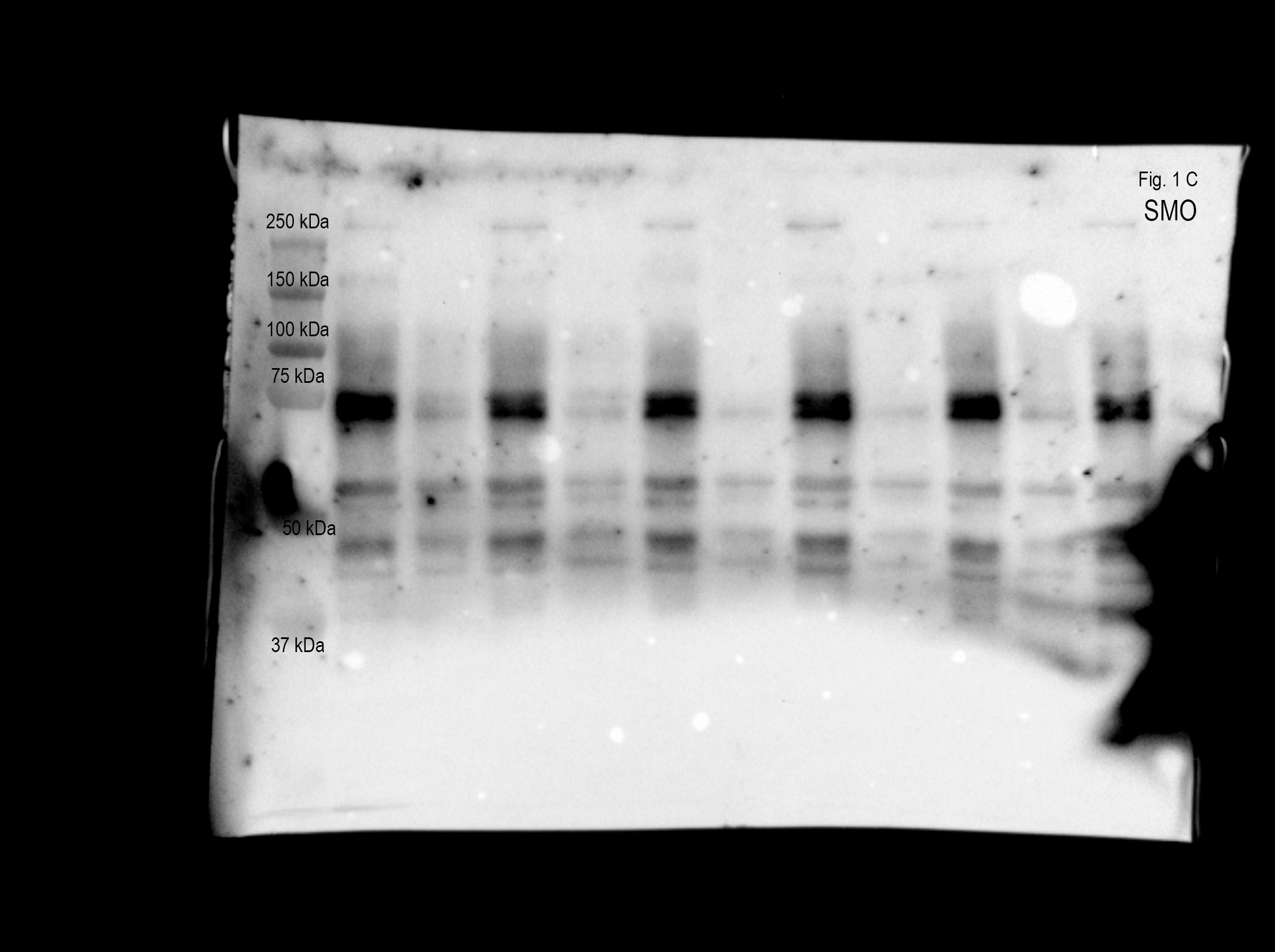

Supplement: Supplementary file 1 [file cancers-14-04776-s001.zip › File S1-blot jpg/2. FIG 1C SKMEL sISMO SMO.jpg]

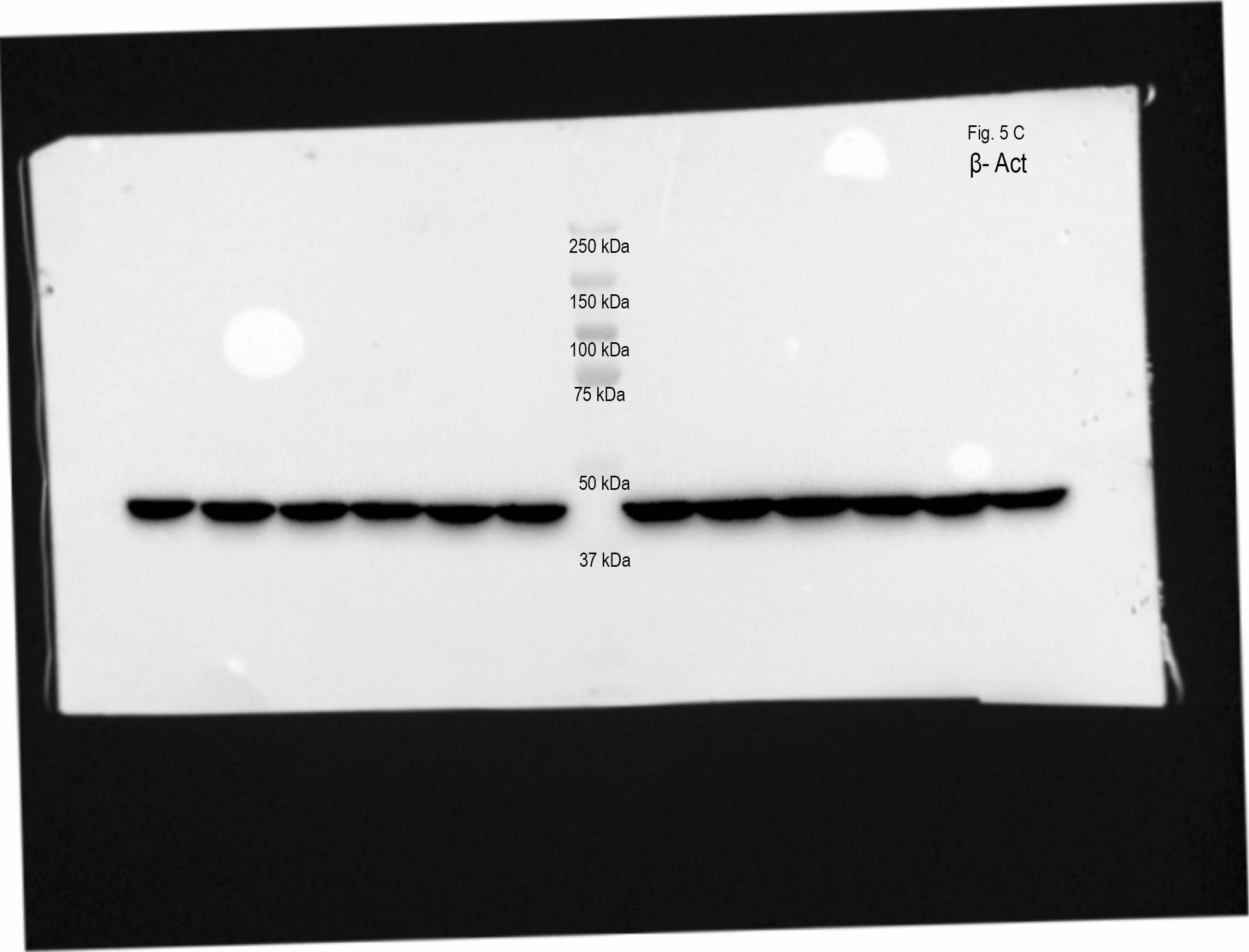

Supplement: Supplementary file 1 [file cancers-14-04776-s001.zip › File S1-blot jpg/20. SKMEL INIBITORI GLI BACT.jpg]

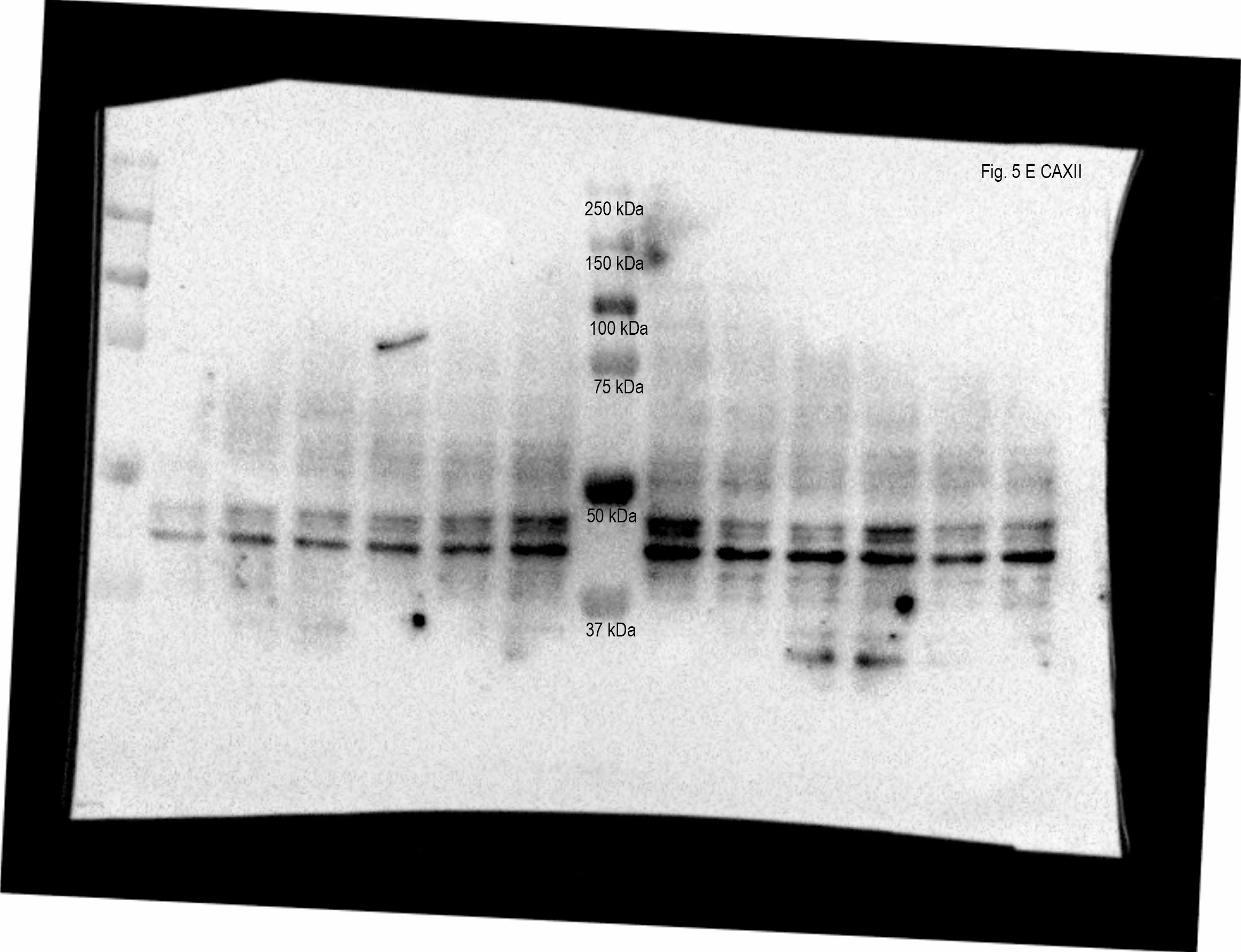

Supplement: Supplementary file 1 [file cancers-14-04776-s001.zip › File S1-blot jpg/21. SKMEL INIBITORI CAXII.jpg]

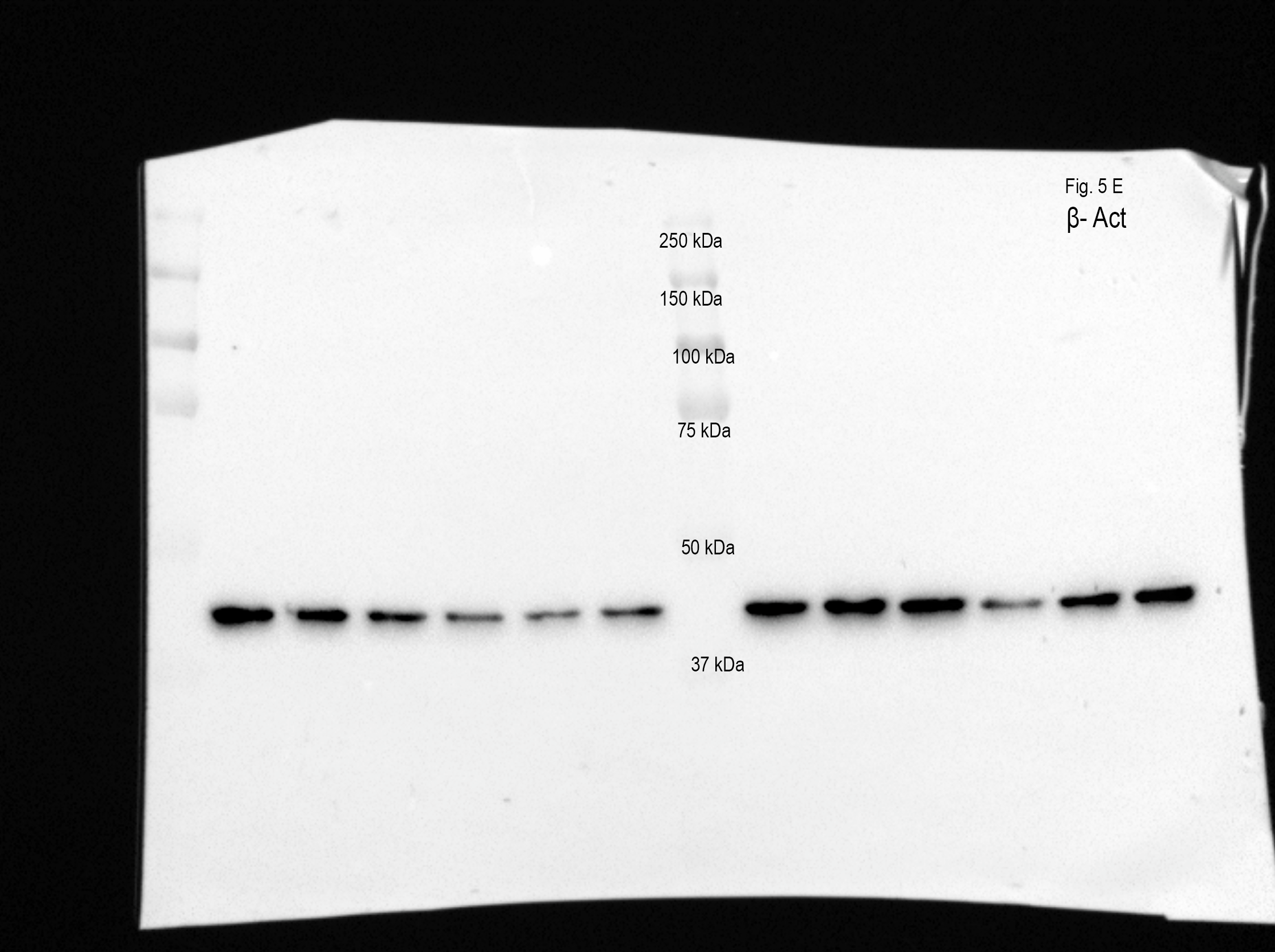

Supplement: Supplementary file 1 [file cancers-14-04776-s001.zip › File S1-blot jpg/22. SKMEL INIBITORI CAXII BACT.png]

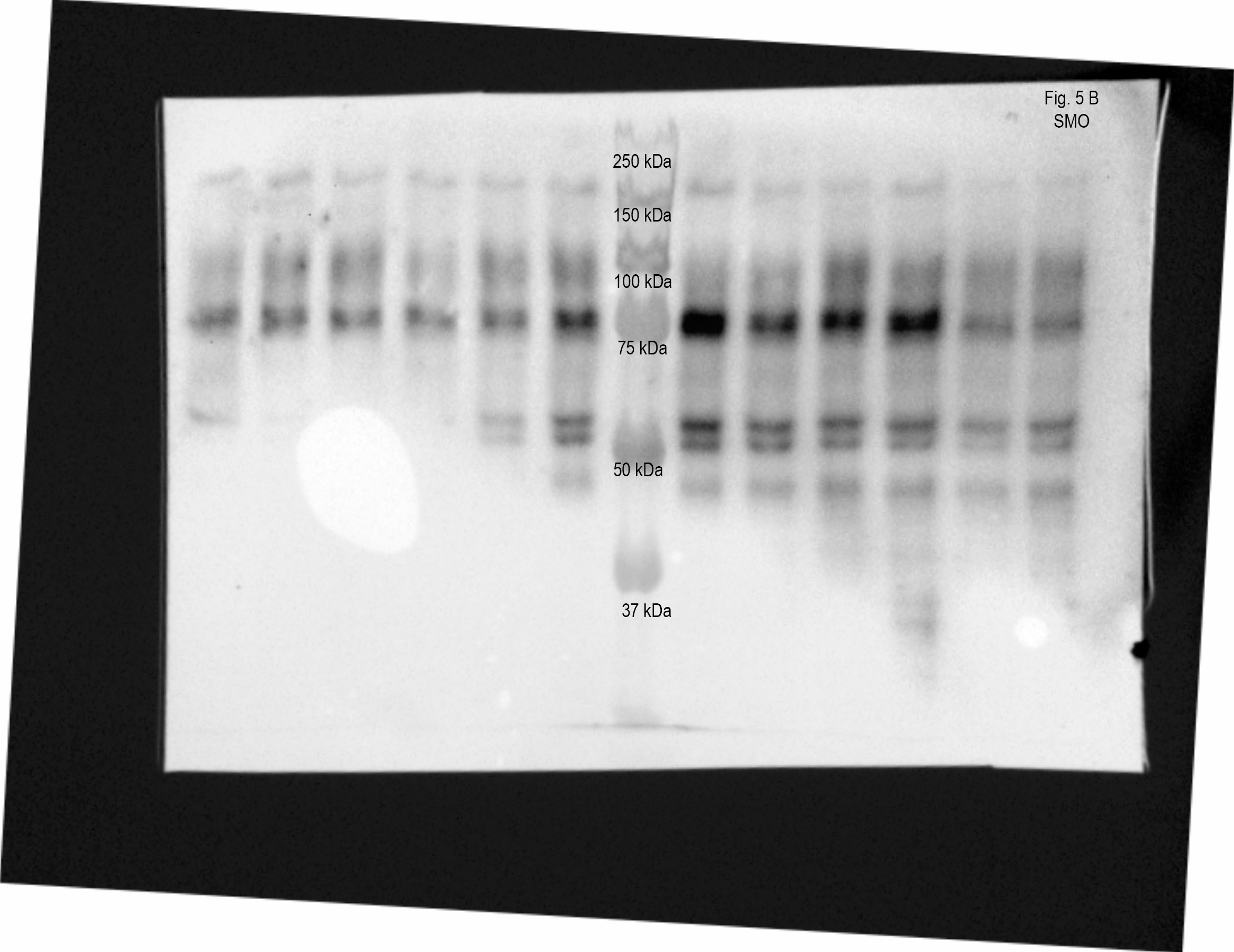

Supplement: Supplementary file 1 [file cancers-14-04776-s001.zip › File S1-blot jpg/23. A375 INIBITORI SMO.jpg]

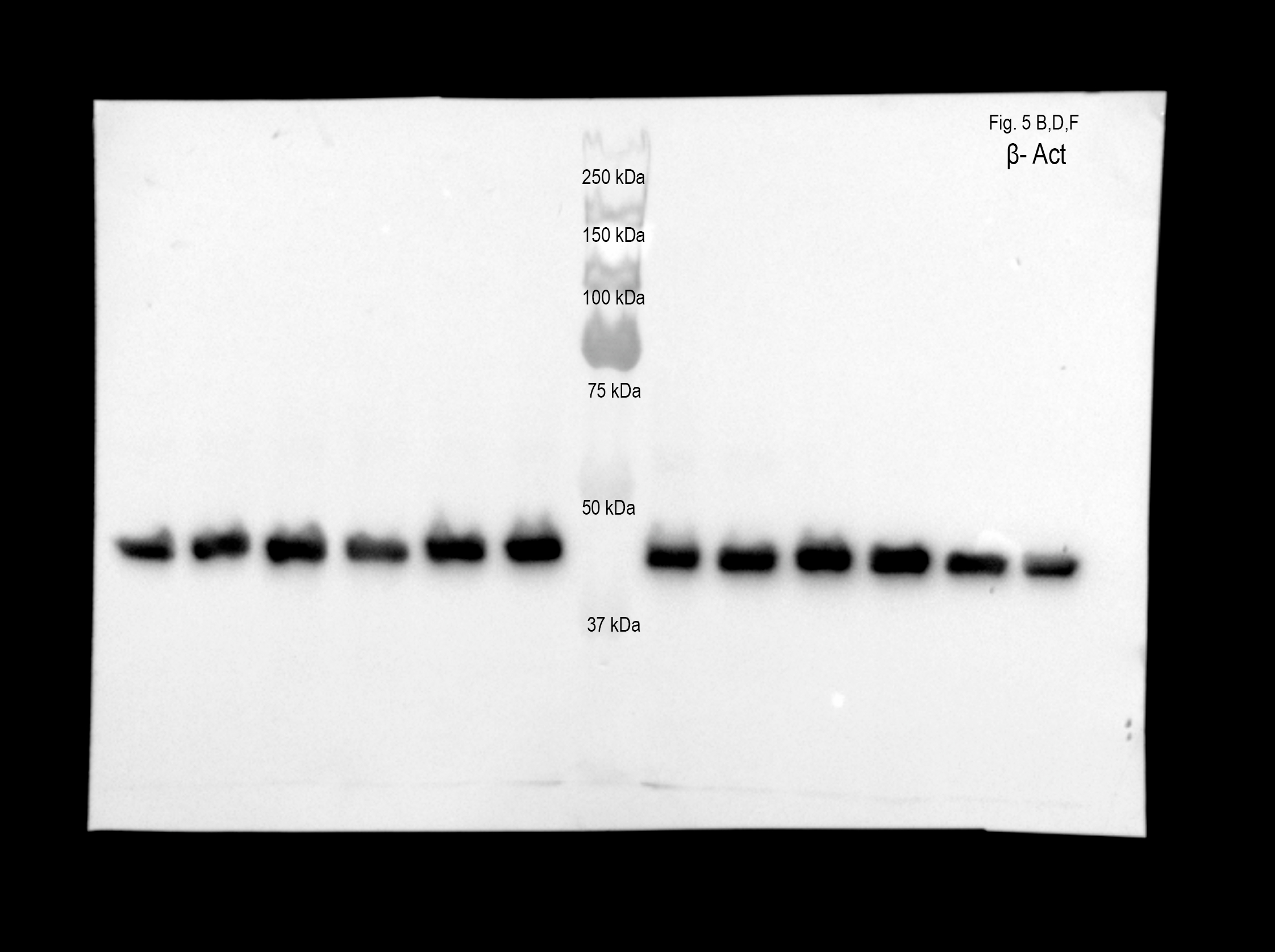

Supplement: Supplementary file 1 [file cancers-14-04776-s001.zip › File S1-blot jpg/24. A375 INIBITORI BACT.jpg]

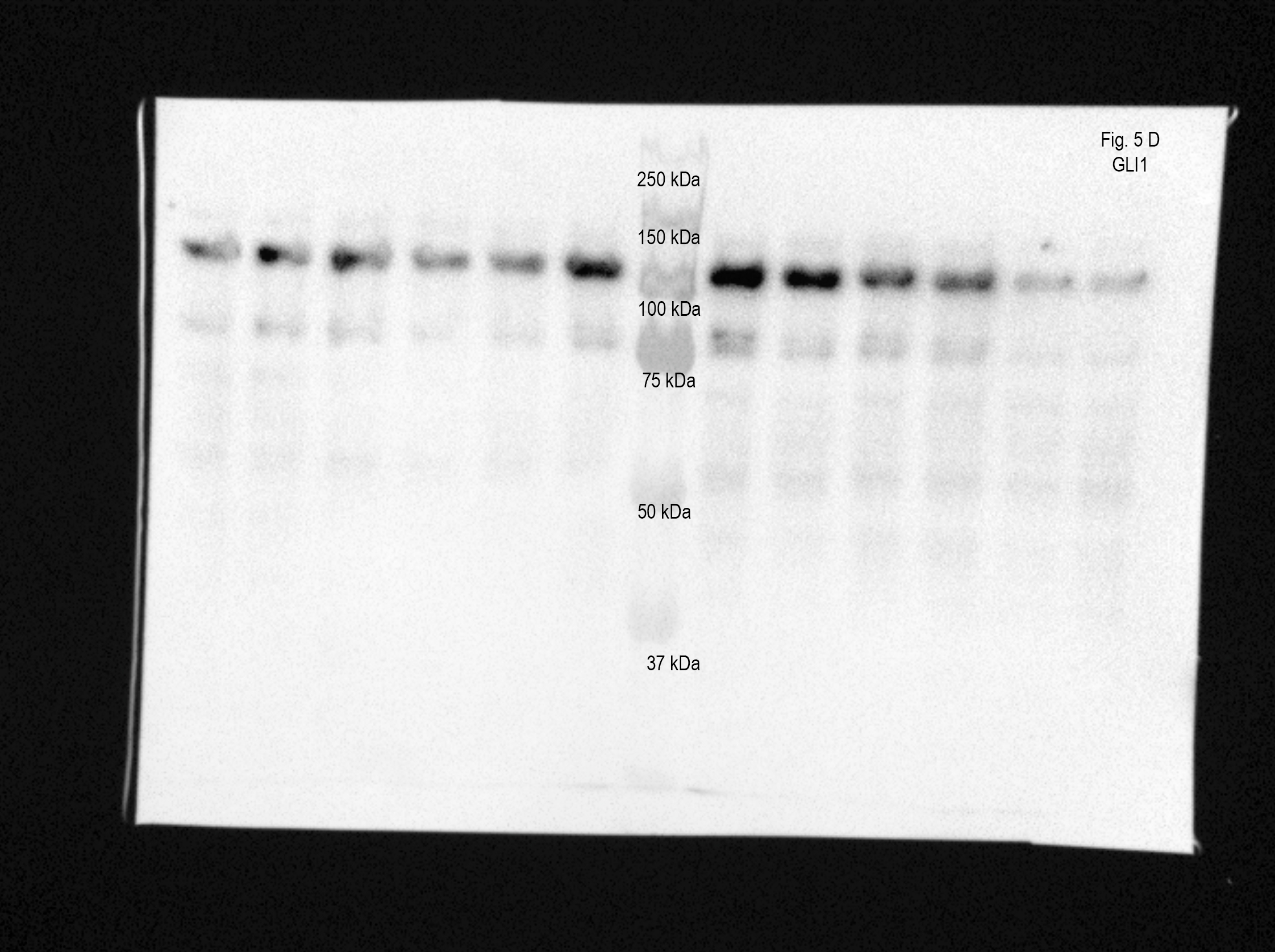

Supplement: Supplementary file 1 [file cancers-14-04776-s001.zip › File S1-blot jpg/25. A375 INIBITORI GLI.jpg]

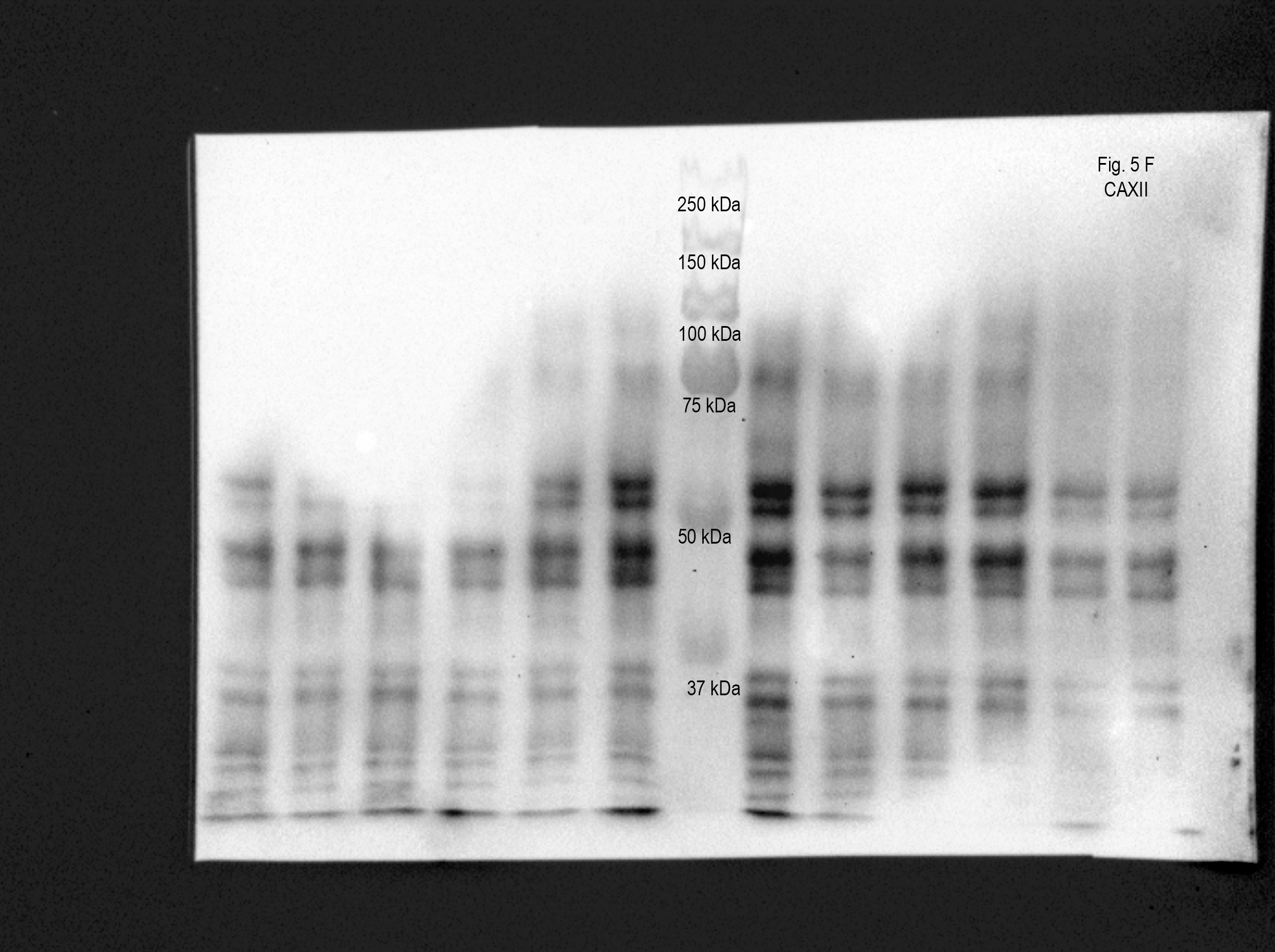

Supplement: Supplementary file 1 [file cancers-14-04776-s001.zip › File S1-blot jpg/26. A375 INIBITORI CAXII.jpg]

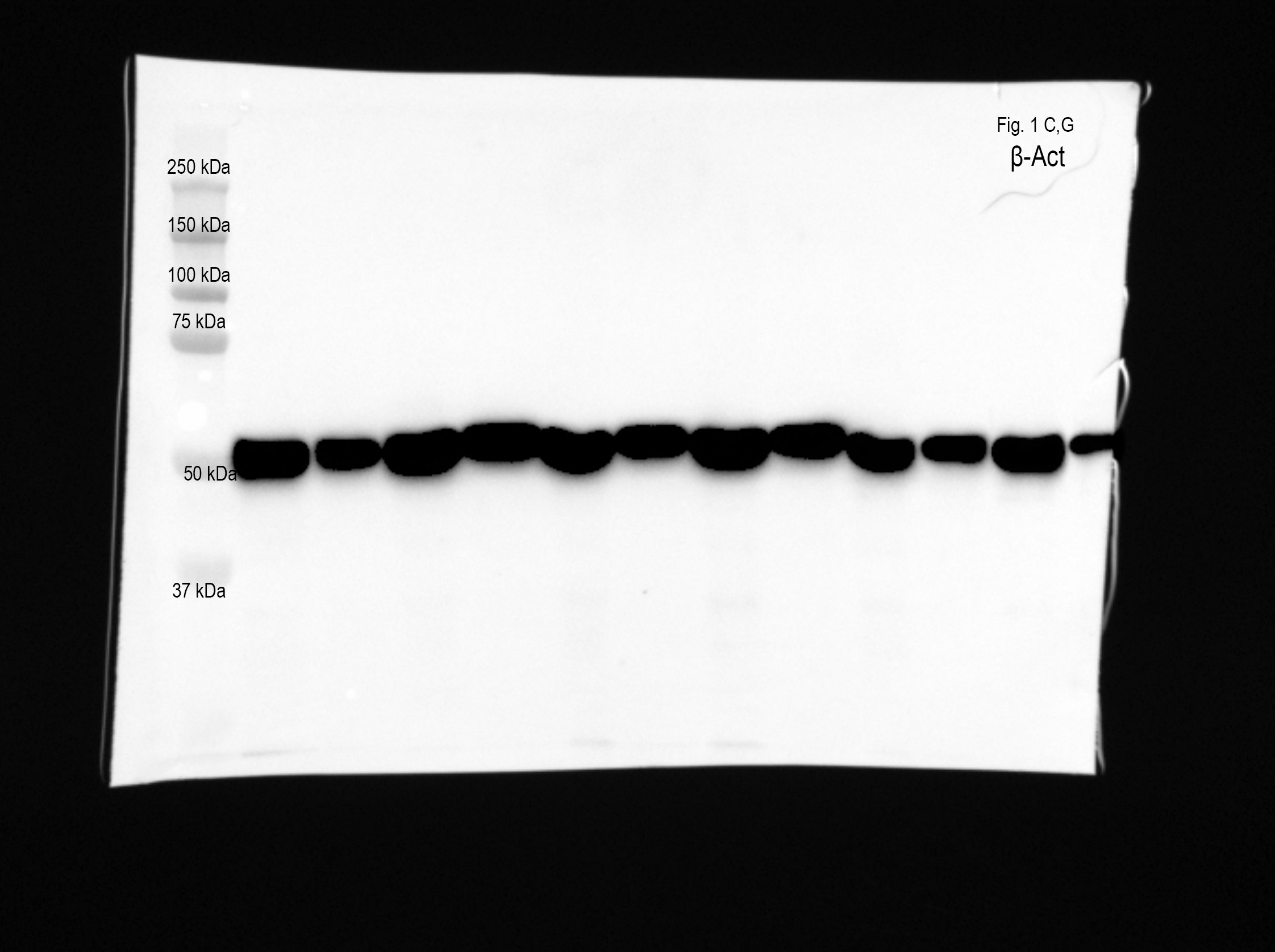

Supplement: Supplementary file 1 [file cancers-14-04776-s001.zip › File S1-blot jpg/3. FIG 1C,G SKMEL sISMO siGLI BACT.jpg]

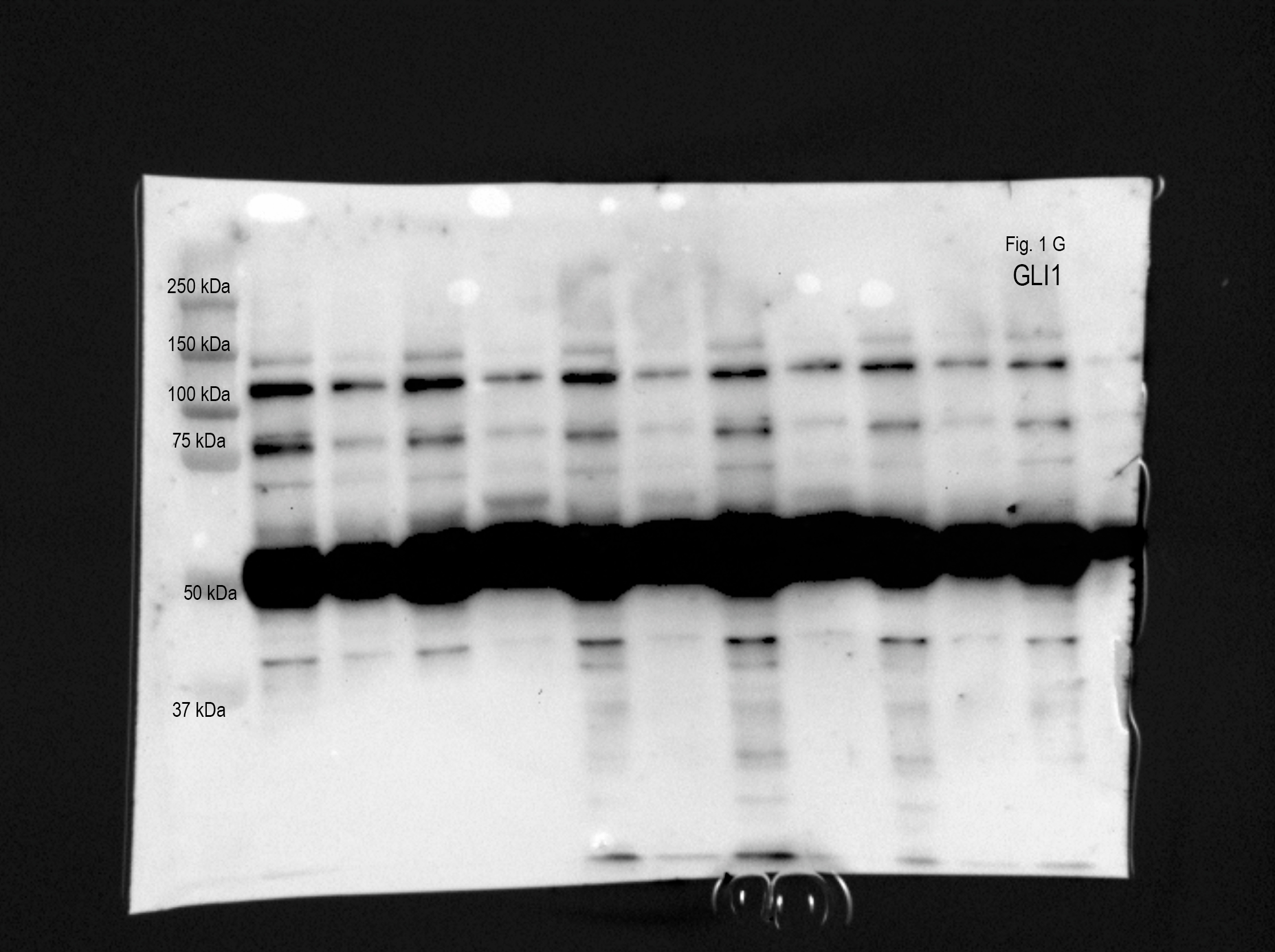

Supplement: Supplementary file 1 [file cancers-14-04776-s001.zip › File S1-blot jpg/4. FIG 1G SKMEL siGLI GLI.jpg]

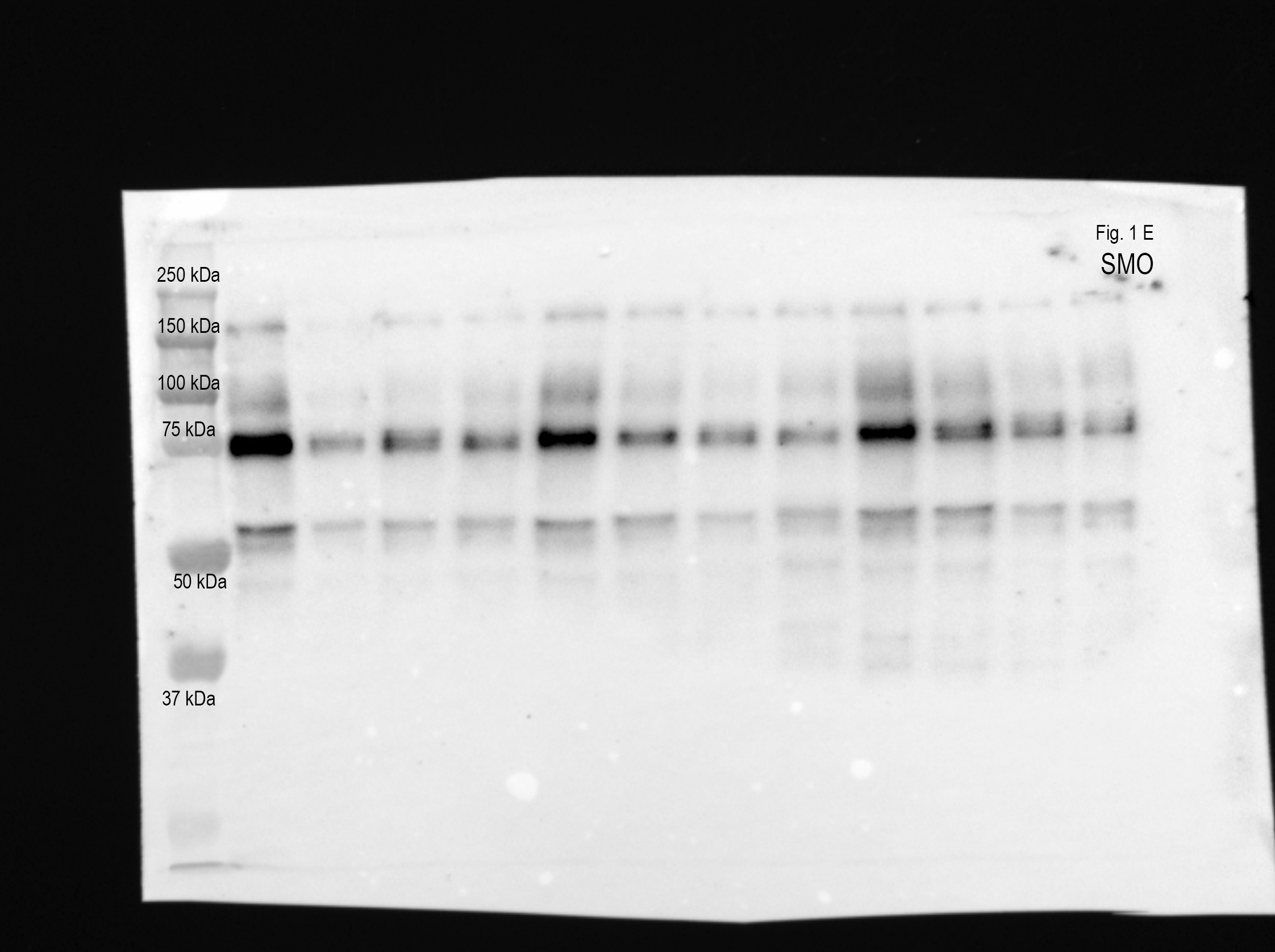

Supplement: Supplementary file 1 [file cancers-14-04776-s001.zip › File S1-blot jpg/5. FIG 1E A375 sISMO SMO.jpg]

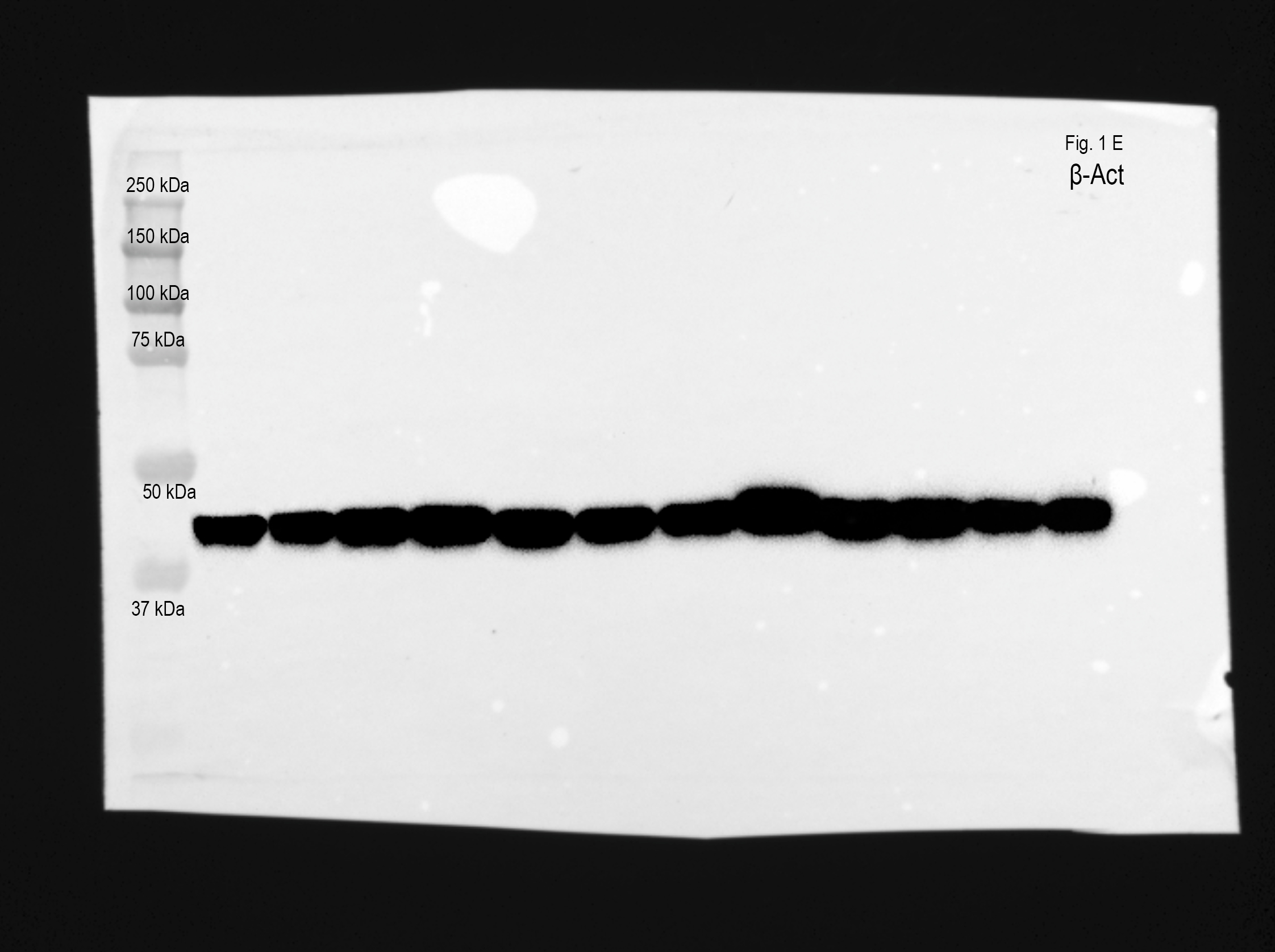

Supplement: Supplementary file 1 [file cancers-14-04776-s001.zip › File S1-blot jpg/6. FIG 1E A375 sISMO BACT.jpg]

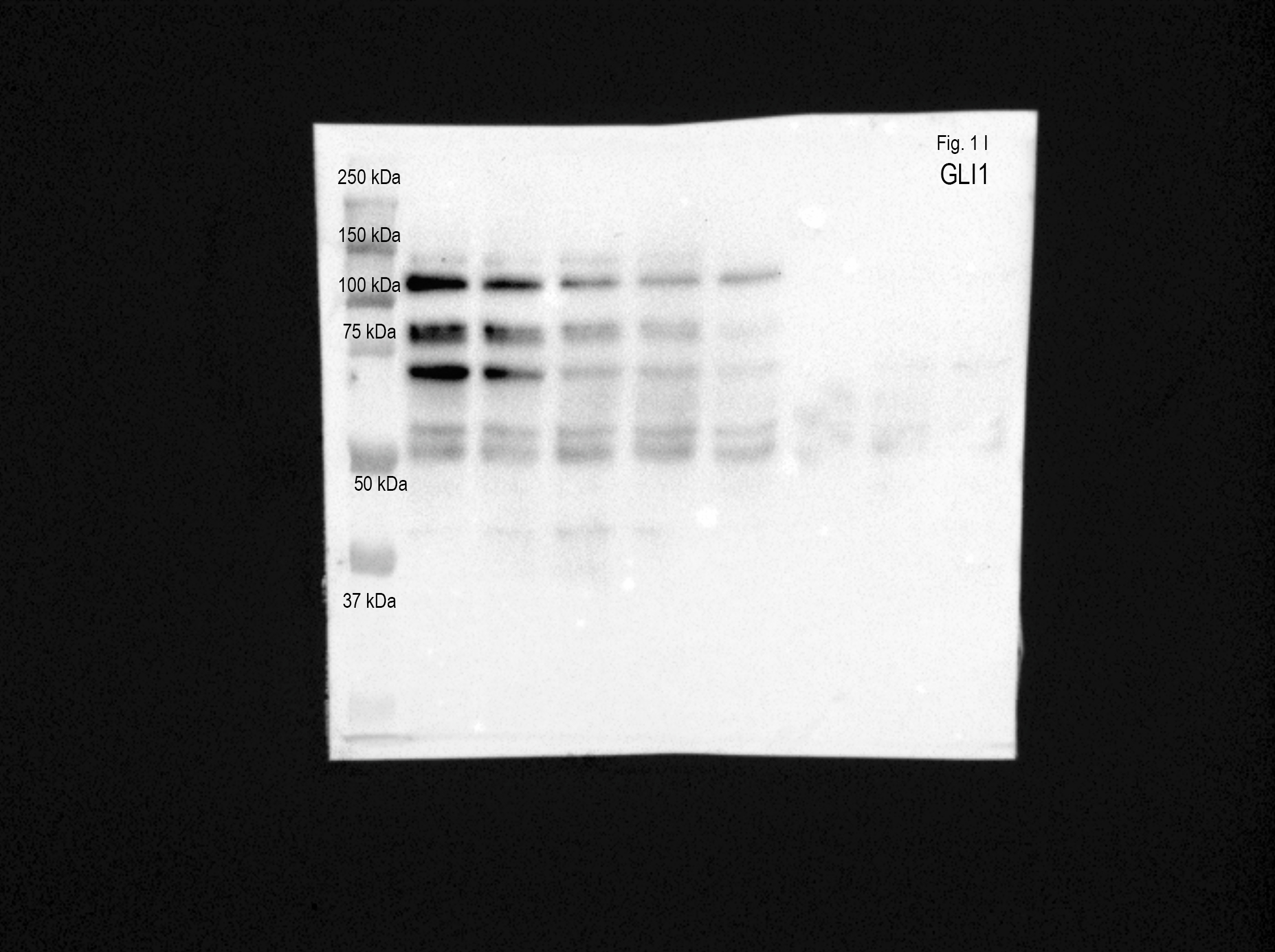

Supplement: Supplementary file 1 [file cancers-14-04776-s001.zip › File S1-blot jpg/7. FIG 1 I A375 siGLI GLI.jpg]

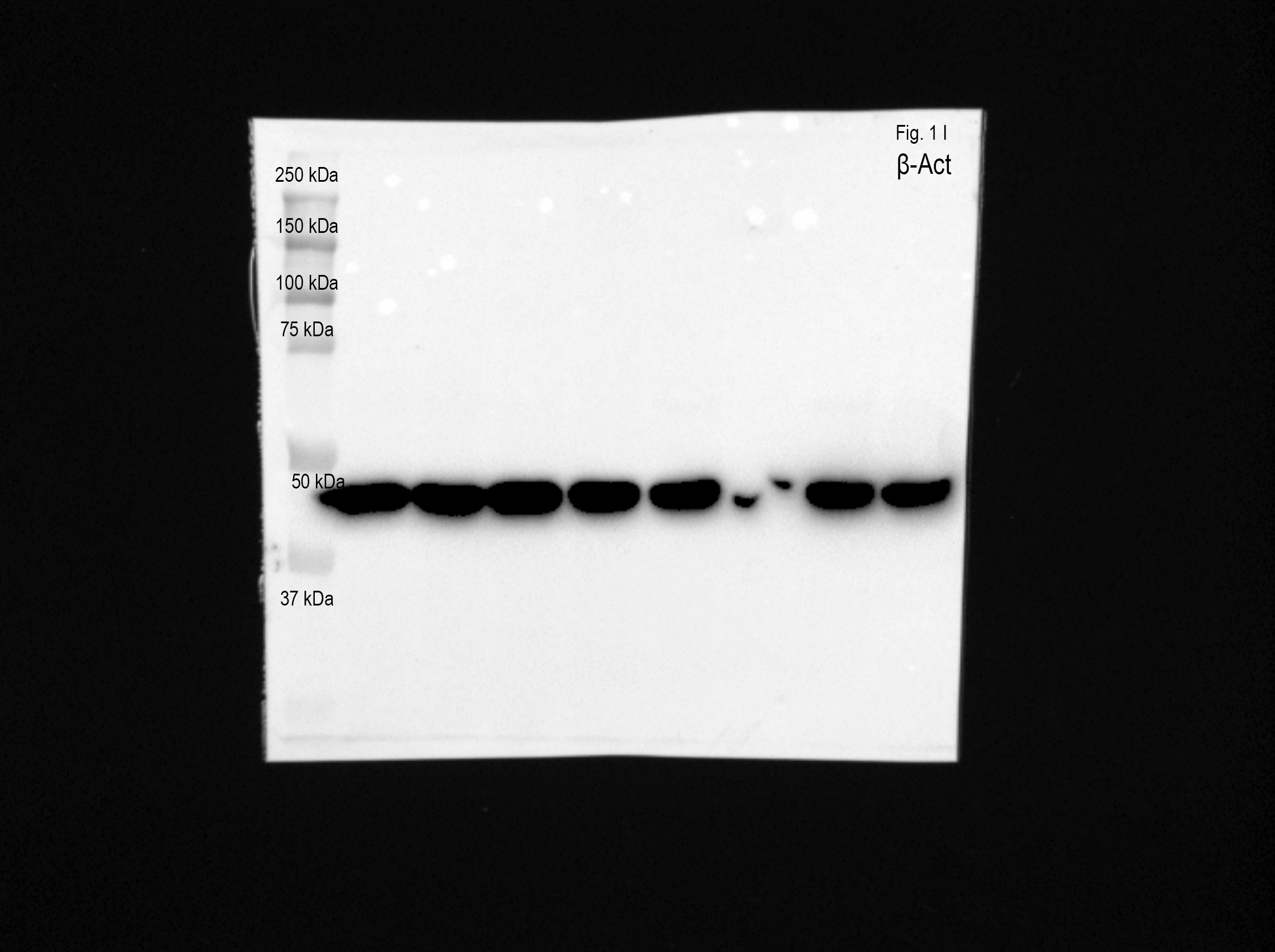

Supplement: Supplementary file 1 [file cancers-14-04776-s001.zip › File S1-blot jpg/8. FIG 1 I A375 siGLI BACT.jpg]

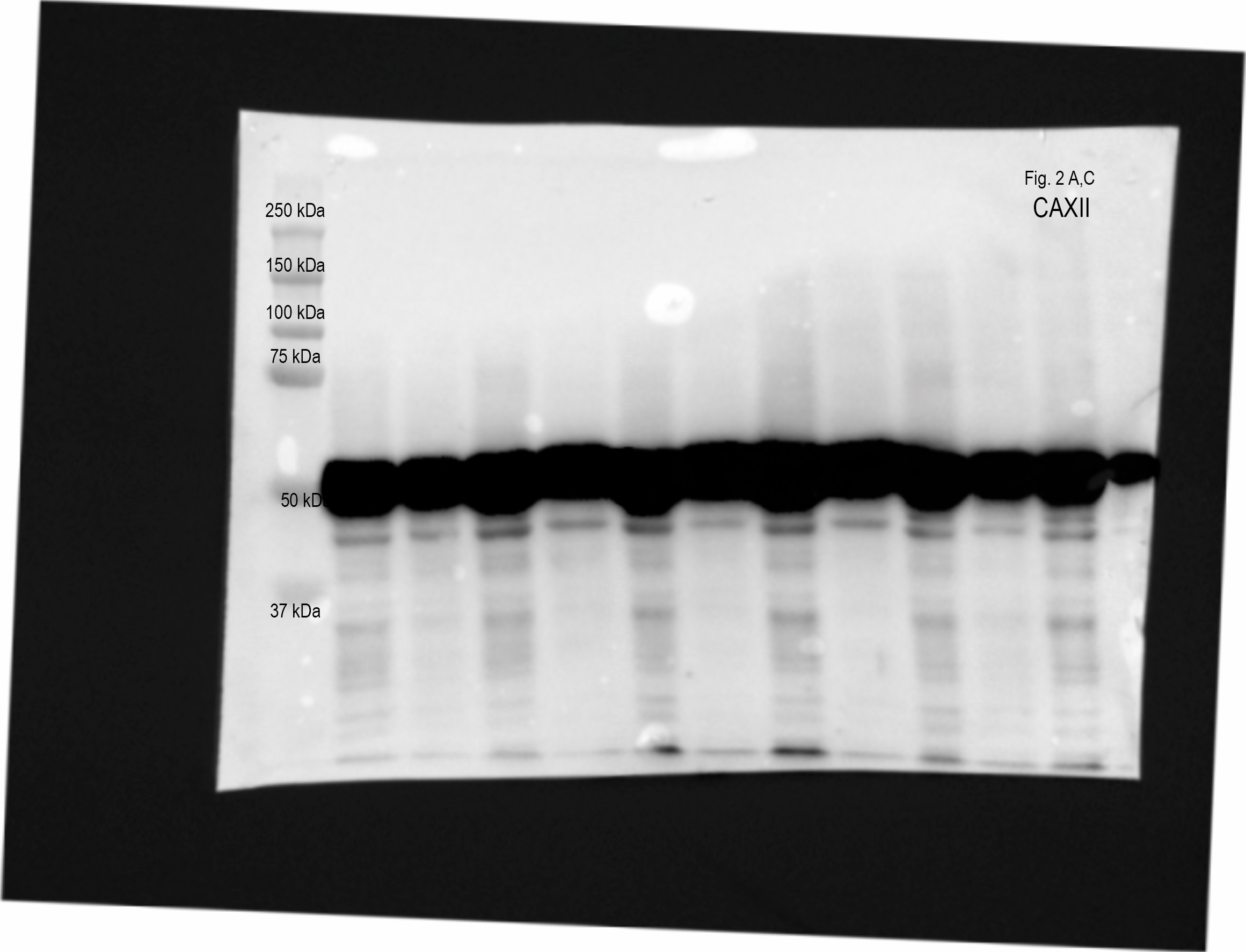

Supplement: Supplementary file 1 [file cancers-14-04776-s001.zip › File S1-blot jpg/9. FIG 2A SKMEL siSMO siGLI CAXII.jpg]
